# Two-segment aging of vestibular perceptual thresholds: motion-specific links to quiet-stance postural sway

**DOI:** 10.3389/fneur.2026.1766090

**Published:** 2026-03-06

**Authors:** Megan J. Kobel, Andrew R. Wagner, Daniel M. Merfeld

**Affiliations:** 1 Department of Speech, Language, and Hearing Sciences, University of Arizona, Tucson, AZ, United States; 2 Department of Physical Therapy, Creighton University, Omaha, NE, United States; 3 Department of Otolaryngology—Head & Neck Surgery, Ohio State University, Columbus, OH, United States

**Keywords:** aging, balance, perception, thresholds, vestibular

## Abstract

**Background:**

Age-related changes in vestibular function may play a role in decreased postural control and increased fall risk. Past research suggests that age-related changes in vestibular perception begin at ~40 years of age; however, this pattern may vary depending on motion trajectory, reflecting contributions of peripheral end-organ structures. Further, motion specific relationships to sway variability have been previously identified. However, relationships between age-related vestibular perceptual changes to multiple sway metrics in multiple planes, reflecting unique aspects of postural control, have yet to be quantified.

**Methods:**

100 healthy adults (21–84 years) completed a vestibular threshold test battery and quiet stance balance assessments. All participants completed motion conditions with predominant contributions from the horizontal canals (2 Hz yaw rotation), vertical canals (2 Hz RALP/LARP tilt), utricles (1 Hz y-translation), saccules (1 Hz z-translation), and integration of canal-otolith cues (0.5 Hz roll tilt). For balance testing, participants completed an instrumented Modified Romberg Balance Test. Regression analyses assessed relationships between age-adjusted measures of vestibular perception to root mean square distance (RMS), mean velocity (MV), and mean frequency (MF) of center of pressure (CoP) in the mediolateral (ML) and anterior–posterior (AP) planes.

**Results:**

Thresholds for most motions - except 0.5 Hz roll tilt - were matched by a two-segment model with stable values below ~40–50 years and linear increases thereafter. For balance conditions with predominant vestibular contributions (i.e., eyes-closed foam-surface), associations between ML RMS to thresholds with predominant contributions from the utricle (y-translation) and canal-otolith integration (roll tilt) were identified. No consistent associations between vestibular thresholds to MV and MF were identified.

**Conclusion:**

Across a population, we were able to confirm that vestibular perceptual thresholds are stable until around middle age after which linear increases in perceptual sensitivity are seen. Our findings linking ML RMS to vestibular perceptual metrics support past hypotheses that sensory noise, as quantified by vestibular thresholds, may contribute to sway variability.

## 1 Introduction

Each year, approximately 28–35% of adults over 65 years of age experience a fall. In older adults, balance and gait impairments have been identified as a principal contributor to falls (1–3) and vestibular dysfunction is a significant contributor to balance and gait impairments (4). However, the vestibular system is complex and consists of multiple end-organs (i.e., canals, otoliths) and contributes to multiple functions, reflecting unique neural pathways. The extent to which specific vestibular pathways contribute to balance dysfunction is incompletely characterized, thus, limiting insight into those at highest fall risk.

Bilateral progressive decline in end-organ function is characteristic of aging in the vestibular system (5, 6) and age-related changes in vestibular functional measures are well-documented (7–10), including measures of vestibular perception (11). Vestibular perceptual thresholds represent the smallest motion stimuli that an individual can correctly perceive, which are typically quantified using direction-recognition tasks (i.e., did I turn left or right?) (11, 12). Previously, a vestibular threshold test battery completed in 105 adults from 18–80 years assessing perception with predominant contributions from multiple vestibular end-organs identified that age-related changes varied in a piece-wise pattern in which thresholds were stable below middle age (~40 years) and then linearly increased, reflecting decreased function, above this age cutoff (13). However, this study has yet to be widely replicated and extended to additional types of motion stimuli. Thus, this study aimed to assess if this two-segment relationship was demonstrated broadly across multiple motion conditions, representing unique aspects of vestibular function. In particular, this was the first study to assess age-related changes for tilts in the planes of the vertical canals [i.e., right-anterior left-posterior (RALP) and left-anterior right-posterior (LARP)] and included a larger sample of adults over 60, who are at increased fall risk and demonstrate increased postural instability (14).

Accumulating evidence also suggests a link between vestibular perception and quiet stance postural control in adults across the age span (13, 15–17). Initial evidence linking vestibular perception to postural control only assessed qualitative pass/fail performance during an eyes-closed standing on foam balance condition (13, 18), which is designed to maximize vestibular contributions to postural control (19). Subsequent studies have assessed these relationships using quantitative performance during instrumented quiet stance balance tasks or posturography (15, 17, 20). Overall, these studies have suggested relationships between variability of postural sway in the mediolateral (ML) plane to thresholds with predominant contributions from the utricle (i.e., y-translation), saccule (i.e., z-translation), and/or canal-otolith integration (i.e., roll tilt) (15, 17, 20).

Associations between vestibular perceptual thresholds and sway variability have been proposed to represent the shared influence of vestibular sensory noise. Vestibular perceptual thresholds serve as an assay of neural noise, which can originate from the periphery or central processing of motion cues (12, 21, 22). Further, models of balance control attribute sway during quiet stance balance tasks to internal noise, which includes sensory noise (19, 23–27). Thus, theoretically, increased vestibular sensory noise (i.e., increased vestibular thresholds) may lead to greater variability in postural sway (15, 17). However, perceptual thresholds also depend on non-sensory factors such as attention, decision processes, and response criteria (12, 28, 29), particularly in psychophysical tasks, which may also show age-related changes. Accordingly, in the present study we interpret threshold–sway relationships as consistent with a sensory-noise framework, while recognizing that other factors may also contribute to between-subject variability in threshold estimates.

During quiet stance, multiple metrics can be quantified, representing independent characteristics of CoP metrics including displacement, velocity, and frequency measures (23). While sway magnitude or displacement is commonly reported, relationships between vestibular perceptual thresholds and sway dynamics (e.g., velocity or frequency) have been less frequently evaluated across a broad adult age range and across multiple vestibular motion types, limiting our understanding of how perceptual performance relates to different aspects of postural behavior. To capture key facets of postural control while limiting multiple comparisons, we specified RMS as the primary outcome *a priori*, as it summarizes sway magnitude/variability. Secondary analyses examined mean velocity (MV), which reflects the overall rate of CoP corrections and is often interpreted as the amount of regulatory activity, and mean frequency (MF), which characterizes the temporal structure of sway dynamics (i.e., how rapidly corrective behavior fluctuates) (30). Together, these outcomes allow us to test whether vestibular perceptual measures relate primarily to sway magnitude and/or also to sway dynamics.

Past findings suggested that age adjustment explained 20.7% of the between subject variation in threshold data (13). Higher or lower thresholds in specific motion conditions may represent an individual trait that underlies the variation in thresholds observed (18). Importantly, age is a strong determinant of both vestibular thresholds and postural sway; therefore, analyses that relate thresholds to center-of-pressure (CoP) metrics can be confounded if age-related covariance is not addressed. A common approach is to include age as a covariate in regression models of CoP outcomes. However, this strategy implicitly assumes that age enters the model in an appropriate functional form (often linear). While sway metrics frequently vary approximately linearly with age over adulthood, emerging evidence indicates that vestibular thresholds may exhibit non-linear age relationships. Under these circumstances, treating age as a single linear predictor can be mis-specified for the threshold component and may obscure whether CoP associations reflect age per se versus individual differences in vestibular perceptual precision.

To address this, we used age-adjusted (model-derived) thresholds that remove the expected age-related component of each motion-specific threshold based on the fitted age–threshold relationship. This approach allows us to test whether between-subject differences in perceptual sensitivity beyond chronological age are associated with postural control, particularly in conditions that depend heavily on vestibular cues (eyes closed on a compliant surface). Therefore, we aimed to: (1) determine whether the relationship between vestibular thresholds and age follow a two-segment model when assessed for multiple motion types; and (2) quantify associations between age-adjusted thresholds and multiple CoP metrics during quiet stance, particularly under vestibular-dependent conditions (i.e., eyes closed, compliant surface). We hypothesized that vestibular thresholds would show age-related changes consistent with a two-segment pattern and that motion-specific differences would emerge, reflecting distinct vestibular end-organ contributions. Further, we hypothesized that higher age-adjusted vestibular thresholds would be associated primarily with greater ML RMS, consistent with vestibular-related variability contributing to ML sway. Associations between age-adjusted thresholds and MV/MF, if present, would be smaller and less consistent than those observed for RMS, reflecting that sway dynamics may be influenced by additional control and strategy factors beyond sway magnitude.

## 2 Materials and methods

### 2.1 Participants

Perceptual thresholds and quiet stance balance tasks were measured in 100 participants (56F/44M; Table 1). Participants were recruited across the age span from 21–86 years of age and recruitment was targeted to provide a representative sample of adults across each decade. As previous data suggests that vestibular perceptual thresholds were stable below 40 years (13), we sampled 40 adults below this age cutoff in order to increase precision of our baseline threshold measures.

**Table 1:** Geometric mean and 95% confidence interval of each threshold for each age group.

Age range	2 Hz Yaw rotation	2 Hz RALP/LARP Tilt	0.5 Hz Roll Tilt	1 Hz y-translation	1 Hz z-translation
20–29 (11F/9M)	0.703 (0.281–1.763)	0.669 (0.288–1.554)	0.790 (0.338–1.850)	0.710 (0.235–2.147)	1.696 (0.732–3.932)
30–39 (9F/11M)	0.607 (0.71–1.358)	0.593 (0.265–1.326)	0.854 (0.408–1.789)	0.563 (0.233–1.360)	1.472 (0.455–4.659)
40–49 (8F/6M)	0.740 (0.284–1.930)	0.813 (0.349–1.894)	0.944 (0.450–1.982)	0.618 (0.269–1.417)	2.083 (0.835–5.195)
50–59 (8F/2M)	0.696 (0.245–1.972)	0.803 (0.361–3.807)	0.934 (9.387–2.254)	0.609 (0.279–1.331)	2.538 (0.878–7.337)
60–69 (10F/5M)	0.880 (0.211–3.679)	1.173 (0.361–3.807)	1.195 (0.517–2.765)	0.863 (0.309–2.404)	3.352 (1.699–6.613)
70–79 (7F/6M)	1.1870 (0.431–3.263)	1.682 (0.610–4.641)	1.579 (0.668–3.734)	1.526 (0.653–3.567)	5.918 (2.086–6.786)
80–89 (3F/5M)	1.307 (0.597–2.859)	1.636 (1.451–1.845)	1.468 (0.674–3.197)	1.981 (0.910–3.220)	5.477 (3.631–10.54)

All participants completed testing for all thresholds. However, 5 participants in the 80–89 age group could not complete RALP/LARP thresholds. Thus, the summary statistics represent only 3 subjects who completed these measures, which may reflect individuals having lowest thresholds.

All participants were community-dwelling and ambulatory without aids and completed a health screening questionnaire prior to enrollment in the study. Subjects were excluded on the basis of reporting: (1) a history of vestibular disorders except history of benign paroxysmal positional vertigo greater than 5 years in the past, (2) neurological disorder (e.g., Parkinson’s disease), (3) recent (<6 month) orthopedic injury potentially impacting balance performance, (4) and other major health conditions which could impact vestibular function and/or balance performance (e.g., cancer, peripheral neuropathy), (5) eye disease or vision worse than 20/40 with correction, and (6) inability to stand or walk unassisted for 15 min. While further demographics information was collected, including height, weight, and medications, these were not included in analyses and only used to confirm equipment size and weight requirements Participation in the study took ~3 h total including breaks. Breaks were given at least once every 30–40 min or sooner if requested. Participants completed three blocks of vestibular thresholds (~30 min each). After the first two blocks, participants took at least a 5-min break and then performed blocks of quiet stance balance testing (~5–10 min).

### 2.2 Vestibular perceptual thresholds

Vestibular perceptual thresholds were quantified using a whole-body direction recognition task. Methodology has been used extensively by our lab (13, 31–35) and others (15, 36–38). The methodology utilized has been recently reported in detail (17, 34, 35), thus, is briefly outlined below.

Participants were seated in a rigid chair attached to a MOOG six degree of freedom (6DOF) motion platform (6DOF2003E, Aurora, NY). Their heads were restrained using a motorcycle helmet which was affixed to the chair. All testing was completed in a light tight room to remove potential visual cues, and potential auditory cues were passively attenuated using insert headphones (~20 dB sound pressure level (SPL)) in addition to passive attenuation provided by the motorcycle helmet. As well, during each motion stimuli, ~60 dB SPL white noise was presented binaurally over the insert headphones to provide active noise masking and to cue participants to a motion stimulus.

Motion stimuli were single cycles of sinusoidal acceleration delivered via the MOOG motion platform. For each threshold measure, 100 trials of a single motion (e.g., yaw rotation) were presented. To optimize efficiency of level of stimulus presentation, an initial 2-down/1-up (2D/1 U) staircase was implemented in which stimulus magnitude was halved after two consecutive correct responses until the first incorrect answer. After this initial staircase, stimuli amplitudes were adjusted using an adaptive four-down, one-up (4D1U) staircase in which step sizes were selected using parameter estimation by sequential testing (PEST) rules (39). After each motion stimulus was delivered, the participants were instructed to indicate perceived direction of motion (e.g., right or left) using handheld buttons. When participants were unsure, they were instructed to make their best guess.

Six unique thresholds were assessed in all participants: (1) 2 Hz yaw rotations with predominant contributions from the horizonal canals (34), (2) 2 Hz right-anterior, left-posterior (RALP) tilts (i.e., tilting from upright 45 degrees offset from midline yielding stimuli forward to the right or backward to the left) with predominant contributions from the vertical canals, (3) 2 Hz left-anterior, right-posterior (LARP) tilts (i.e., tilting from upright 45 degrees offset from midline yielding motions forward to the left or backwards to the right) with predominant contributions from the vertical canals (33, 34), (4) 0.5 Hz head-centered roll tilt (i.e., tilting from upright to the right or to the left in the coronal plane) targeting canal-otolith integration (33, 40), (5) 1 Hz inter-aural y-axis (“y-translations”) with predominant utricular contributions, and (6) 1 Hz superior–inferior *z*-axis (“z-translations”) with predominant saccular contributions (35, 41).

For all motions except 0.5 Hz roll tilt and RALP/LARP tilts, after each trial and subsequent subject response, the next trial began from the final displacement of the previous trial. For RALP/LARP tilt, subjects responded while still tilted prior to returning to upright. For roll tilt, participants were returned to upright prior to response to minimize static otolith cues (34). We previously found that thresholds were not significantly different between this paradigm and the approach used for RALP/LARP tilts in which participants provided response prior to their return to upright (33). For all motions, a minimum of 3 s was provided between trials or between return to upright and next trial in order to reduce the impact of motion aftereffects (42, 43).

For each vestibular threshold, binary responses and stimuli magnitudes were fit with a Gaussian cumulative density function using a maximum likelihood estimate and a bias-reduced generalized linear model (*brglm,* MATLAB 2023a) (44). A delete one jackknife method was implemented in which the psychometric function was fit to all trials and then re-fit repeatedly with each trial omitted in turn, flagging trials whose removal produced a disproportionately large change in fit quantified by the delta-deviance (Δdeviance; treated as χ^2^ with 1 df, *p* < 0.01) for potential exclusion (45). The majority of trials had no identified lapses (79.8%; *n* = 471), while 16% (*n* = 95) of all trials had 1 identified lapse, and only 3.3% (*n* = 20) had 2 lapses identified. There was no significant impact of age on lapse rate for any threshold condition (*z* = 0.45, *p* = 0.654). The threshold parameter (σ, i.e., the psychometric width) corresponds to a stimulus magnitude which would on average yield an accuracy of 84.1% (12, 44).

In 12 participants total, aged 70 or older, RALP and/or LARP tilt threshold tests were discontinued as participant responses yielded stimulus values which were over the displacement limits of the device. In 5 subjects, this happened for both RALP and LARP tilt thresholds prior to completing 50 trials. Additionally, seven other participants were unable to complete the full 100 trials of either RALP or LARP tilt thresholds. Across the 88 participants with both RALP and LARP tilt thresholds, RALP thresholds and LARP thresholds were not significantly different from one another [*t*(89) = −0.112, *p* = 0.911] and were strongly correlated (*r* = 0.805, *p* < 0.0001). Thus, the average of RALP tilt and LARP tilt thresholds was included in analysis for 88 participants. Of those that were only able to complete either RALP or LARP without increasing stimulus levels above motion platform, the threshold which was completed was included in analyses (*n* = 7). Analyses were completed excluding these subjects and overall conclusions were not meaningfully different, thus were included in order to enable analysis of these older adults who presented the highest thresholds. This yielded 5 total older adults with missing RALP/LARP tilt thresholds.

### 2.3 Quiet stance balance

Participants completed five quiet stance balance tasks: Condition (1) eyes open (EO) on a firm surface, Condition (2) eyes closed (EC) on a firm surface, Condition (3) EO on an Airex foam surface, Condition (4) EC on an Airex foam surface, and Condition (5) EC on Memory foam compliant surface. A standard Airex closed cell foam low density foam pad (Switzerland; 6 cm thickness) was used in addition to a medium density memory foam pad (SunMate, Leicester, North Carolina). This memory foam pad was also used in the National Health and Nutrition Examination Survey (NHANES) (4) and in our past study relating thresholds to “pass/fail” balance performance (13, 18). Both pads were used due to our pilot data suggesting increased test difficulty (i.e., larger changes in quantitative balance metrics relative to baseline performance) using memory foam. This allowed us to provide a greater postural control challenge, and potentially further disrupt proprioceptive cues for balance to emphasize vestibular contributions to balance performance.

In all conditions, stance was maintained for 67 s. Participants stood with their feet positioned so the medial borders were touching and with arms crossed at the chest. Center of pressure (CoP) data were collected at 100 Hz from a triaxial AMTI force plate (Watertown, MA). To remove transient responses, the first 7 s of data were removed in all analyses. Raw CoP data from each trial were low pass filtered with a 25 Hz cutoff using a 4th order zero-phase-lag filter (*filtfilt,* MATLAB v2020a, Natwick MA) as previous data suggests acceptable within subject variability with these filter settings applied to quiet stance data (46). Our primary metric of interest was mediolateral (ML), and anterior–posterior (AP) root mean square distance (RMS) which is equivalent to the standard deviation of the zero-mean CoP tracing (30). We chose to focus our analyses on RMS as our past data suggested strongest relationships between thresholds and this parameter (17, 20).

We also assessed ML and AP mean velocity (MV) and mean frequency (MF) as secondary outcome measures of interest as it has previously been shown that MV and MF are not strongly correlated with RMS or each other (23). MV represents the average instantaneous velocity of the CoP and MF represents the rotational frequency of the CoP which is proportional to the ratio of the mean velocity to the mean distance of displacement (30). Our past data in a small sample of young healthy adults (*n* = 20) suggested a potential relationship between ML MV to 0.5 Hz roll tilt thresholds (20) and others have identified changes in MV with aging (14, 47). Previously, we failed to identify significant associations between MF to roll tilt thresholds in young adults; however, we elected to include this in analyses as this represents a unique parameter characterizing postural control (23) and past evidence suggests that MF, particularly when assessed in the AP plane, may differentiate fallers from non-fallers (48–50).

### 2.4 Data analysis

All thresholds were log transformed as data exhibited a lognormal distribution (Shapiro–Wilk, *p* > 0.08) consistent with past reports (13, 51, 52). Geometric means are therefore reported, and calculations were performed using logarithmic data. Parametric analyses were completed to assess a potential impact of sex. Pearson correlation was completed to test for correlation between thresholds in different axes. All statistical analyses were performed using Stata (v. 18; College Station, TX).

#### 2.4.1 Vestibular thresholds and aging

Past evidence suggests that vestibular perceptual thresholds vary with age in a two-segment manner in which there is a flat plateau below an age cutoff (~42 years) and linear increases in thresholds are identified after this cutoff (13). We fit two-segment linear models which largely mirrored original methodology in order to verify this pattern of age-related changes. These past efforts by Bermudez-Rey et al., performed fitting across all thresholds in addition to for each threshold individually; however, we only performed fits for each threshold as visual inspection of our data suggested differences between threshold conditions, particularly for z-translation (discussed more below).

For each motion direction, the population threshold data were fit with a two-segment linear model having three parameters: (1) a “baseline” parameter, which captures the average threshold for adults below the age cutoff, (2) an “age cutoff” parameter above which thresholds increased, and (3) a “slope” parameter representing age-related changes in thresholds occurring after the identified age cutoff. Additionally, simple linear regression models were fit to each threshold with two parameters: (1) an intercept, and (2) a slope representing age-related changes across the age span. Goodness-of-fit of the proposed two-segment models in comparison to simple linear regression models were assessed with adjusted R^2^, likelihood ratio tests, Akaike Information Criterion (AIC), and Bayesian Information Criteria (BIC). All fits were performed using a nonlinear least squares estimation (*nl,* Stata v. 18.0) All parameters were transformed after fits to present data in the original physical units rather than log-transformed data.

#### 2.4.2 Relationships between CoP metrics and vestibular thresholds

In order to characterize the relationships between age-adjusted thresholds to CoP metrics including RMS, MV, and MF, several regression models were assessed. First, full regression analyses across all subjects between all model-derived age-adjusted thresholds to AP and ML RMS, MF, and MV of the CoP.

All adjustments were performed using the motion-specific parameters derived from the two-segment linear model described above. For subjects younger than the age-cutoff, age-adjusted thresholds were raw thresholds measures. For subjects older than the age-cutoff, age-adjusted thresholds were calculated using the equation(s). Adjustments were performed in the log domain and transformed back to physical units for ease of interpretation.


$$\begin{array}{l} \mathrm{age}\;\text{adjusted thresholds}=\text{threshold}*[\text{baseline}/\\ \left(\text{slope}*[\mathrm{age}-\text{cutoff}]+\text{baseline})\right] \end{array}$$

$$\begin{array}{l} \mathrm{age}\;\text{adjusted thresholds}\\ =e^{\log(\text{threshold})-\log(\text{slope}*[\mathrm{age}-\text{cutoff}]+\text{baseline})+\log(\text{baseline})} \end{array}$$

Age-adjusted thresholds were selected for primary analyses as age is a strong determinant of both vestibular thresholds and postural sway, and our primary goal was to test whether individual differences in thresholds beyond age relate to sway metrics. Conceptually, this is analogous to using age-residualized predictors and reduces interpretational ambiguity that can arise when age and thresholds covary strongly in the same regression model. We previously published results assessing age included as a linear predictor in a subset of these participants (*n* = 52), and we refer interested readers to this companion article for pertinent figures and analyses (17). Five participants, representing the highest threshold values, had incomplete datasets as they were unable to complete RALP/LARP thresholds. Therefore, individual univariable regression models between each CoP metric and each age-adjusted threshold measure were completed in order to enable inclusion in regression analyses. Multiple-comparison control was applied within pre-defined families: for each CoP metric × plane, we corrected across all threshold tests within the primary vestibular-dependent conditions (Conditions 4–5; *m* = 2 conditions × 5 motions = 10) and within secondary conditions (Conditions 1–3; m = 3 × 5 = 15). Within each family, *p*-values were adjusted using Bonferroni correction (p adj = p × m,), and statistical significance was defined as adjusted *p*-value < 0.05. As vestibular thresholds are correlated, Bonferroni is conservative; we selected this approach to prioritize control of family-wise error for our pre-specified primary hypotheses.

For both full and univariable regression analyses, our primary analyses focused on eye-closed-foam conditions, both Airex and memory (Conditions 4 and 5), due to the predominant vestibular contributions. Secondary analyses assessing the remaining balance conditions (i.e., Conditions 1, 2, and 3), and each threshold were also completed and presented.

Additional secondary analyses were completed using non-adjusted thresholds in the log domain for both full regression and univariable regression analyses. These are presented alongside age-adjusted results in all tables outlining regression results to allow insights into the impact of age-adjustments. For all analyses, regression assumptions were evaluated and screened for influential observations using standard diagnostics, including DFBETAS (threshold = 2/√n), and found no influential data points or meaningful violations that would alter interpretation of the results.

## 3 Results

### 3.1 Overview of thresholds and interrelationships

Consistent with past findings (13), we did not identify a significant difference between males and females across all thresholds using a repeated-measures ANOVA [*F*(1,678) = 0.38, *p* = 0.706]. Additionally, when assessing each motion condition separately, one-way ANOVAs failed to identify a significant effect of sex (all *p* > 0.198). Sex was therefore not included in subsequent analyses.

Pearson product–moment correlations between log-transformed thresholds suggested that all thresholds were significantly and moderately correlated with each other (Bonferroni-corrected *p* < 0.003; Supplementary Table 1). For age-adjusted thresholds, all thresholds displayed weak but significant correlations with each other (*p* < 0.016; Supplementary Table 1), with the exception of the z-translation age-adjusted threshold, which was not significantly correlated with any other age-adjusted threshold.

### 3.2 Results of age-fitting

**Figure 1:**
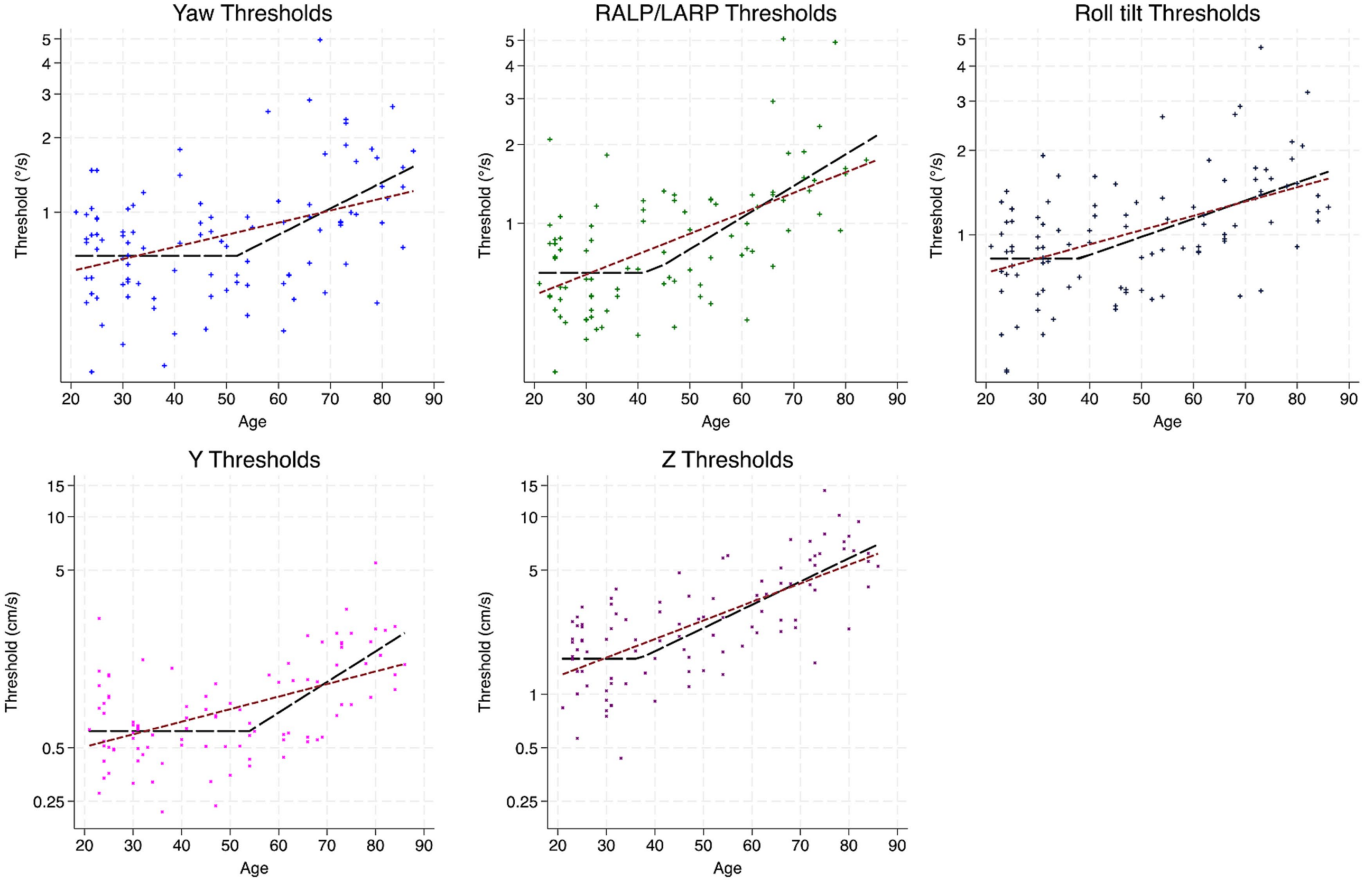
Threshold data for all subjects versus age for each motion condition. Black dashed lines indicate results from two-segment fits while maroon dotted lines indicate fits from simple linear regression.

Geometric mean thresholds for each motion condition per decade are in Table 1. Overall, each motion condition showed an increase in threshold with age suggesting degraded vestibular perceptual precision with age. Parameters obtained from two-segment fits performed for each motion condition separately are in Table 2. Slopes are represented as change per decade in order to present data as a more meaningful unit of change. Results depicting two-segment fits and linear fits are displayed in Figure 1.

**Table 2:** Parameter fits and the associated 95% confidence intervals determined by fitting two-segment functions (see text for details) and linear model (last column).

	Age cutoff	Baseline	Two-segment slope (per decade)	Linear slope (per decade)
Yaw rotation (deg/s)	52 (47–66)	0.67 (0.59–0.76)	0.243 (0.070–0.401)	0.111 (0.062–0.164)
RALP/LARP tilt (deg/s)	42 (32–52)	0.64 (0.57–0.74)	0.278 (0.160–0.394)	0.180 (0.127–0.231)
Roll tilt (deg/s)	38 (30–55)	0.82 (0.72–0.94)	0.149 (0.071–0.227)	0.117 (0.077–0.158)
y-translation (cm/s)	54 (45–62)	0.62 (0.55–0.71)	0.373 (0.215–0.531)	0.151 (0.101–0.203)
z-translation (cm/s)	36 (29–47)	1.59 (1.36–1.85)	0.312 (0.227–0.405)	0.247 (0.198–0.296)

The youngest age-cutoff was seen for z-translation (36.81 years). Both yaw rotation and y-translation thresholds displayed age cutoffs in the early 50’s, 52 and 54 years, respectively, while roll-tilt and RALP/LARP tilt thresholds displayed an age cutoff close to 40 years of age.

Diagnostics comparing fits between linear models and two-segment models are shown in Table 3. Likelihood ratio testing was used to assess nested models (two-segment and linear regression) and results suggested that two-segment models were better fits of the dataset (*p* ≤ 0.037) for all threshold conditions except for 0.5 Hz roll tilt thresholds (*p* = 0.425), where the two-segment fits and linear fits yielded roughly equivalent AIC and BIC values. Overall, the largest improvement in model fit for the two-segment model in comparison to the simple linear regression was noted for y-translation which showed ~15 point decrease in BIC. Changes in BIC greater than 10 are considered “strong evidence” that the model with the lower BIC is a better fit (53) Although likelihood ratio tests favored the two-segment model for several motions (*p* < 0.05), information-criterion changes indicated that meaningful improvement in fit was primarily evident for y-translation (ΔBIC ≈ 15), while yaw rotation, RALP/LARP tilt, and z-translation showed small improvements (ΔBIC < 1), and roll tilt showed no advantage for the two-segment model.

**Table 3:** Diagnostics comparing linear regression models to two-segment fit models (see text for model fit details).

Model	DoF	Adjusted R^2^	Log likelihood	AIC	BIC	Likelihood ratio
Yaw rotation 2 Hz						
Two-segment	3	0.198	−74.83	149.53	157.361	**6.13 (*p* = 0.013)**
Linear	2	0.156	−71.77	153.67	157.362	
Roll tilt 0.5 Hz						
Two-segment	3	0.240	−52.01	109.03	113.24	0.64 (*p* = 0.425)
Linear	2	0.243	−51.69	108.39	117.20	
RALP/LARP tilt 2 Hz						
Two-segment	3	0.367	−55.35	112.36	119.86	**4.35 (*p* = 0.037)**
Linear	2	0.343	−53.18	114.72	119.72	
y-translation 1 Hz						
Two-segment	3	0.272	−64.80	142.08	149.90	**18.87 (*p* < 0.001)**
Linear	2	0.391	−73.38	158.95	164.16	
z-translation 1 Hz						
Two-segment	3	0.506	−65.72	142.21	150.07	**4.66 (*p* = 0.031)**
Linear	2	0.488	−68.98	144.86	150.02	

Values which are statistically significant (i.e. *p* ≤ 0.05) are bolded.

### 3.3 CoP measures

All participants completed quiet stance balance testing; however, data from two participants (1 male from the 40–49 age range, 1 male from the 60–69 age range) were discarded due to equipment malfunction during testing. Thus, 98 datasets were included in balance analyses. In the instance that participants were unable to complete a condition, only data from the incomplete trial was removed while all other conditions were included in analyses. Overall, inability to complete balance testing for any condition was only seen in adults 60 years of age or older while all adults younger than age 60 completed all conditions for the full 67 s. All subjects across all ages were able to complete Condition 1 & 2 (EO and EC on firm surfaces) and three out of 36 adults above the age of 60 were unable to complete Condition 3 (EO on Airex). For our two balance conditions with primary vestibular contributions, 5 participants could not complete EC on Airex (Condition 4), and 11 participants could not complete EC on Memory Foam (Condition 5).

Mean and standard deviations of RMS, MF, and MV across each age group in both the ML and AP planes are presented in Supplementary Tables 2–7 for all balance conditions. A significant main effect of age was noted for ML RMS (*z* = 5.60, *p* < 0.001), AP RMS (*z* = 4.08, *p* < 0.001), ML MV (*z* = 7.78, *p* < 0.001), AP MV (*z* = 7.18, *p* < 0.001), ML MF (*z* = 5.35, *p* < 0.001), and AP MF (*z* = 5.43, *p* < 0.001). A significant main effect of Condition was noted for all metrics (RMS, MF, MV) in both planes (*p* < 0.001). For all measures, all conditions were significantly different from each other (*p* ≤ 0.001).

### 3.4 Relationships between age-adjusted thresholds and CoP

We emphasize that this section (3.4) presents relationships between age-adjusted thresholds and CoP; the following section (3.5) presents relationships between non-adjusted thresholds and CoP.

To help orient the reader to what follows, we note that the following subsections (i.e., 3.4.1, 3.4.2, and 3.4.3) show that in our primary conditions (EC Airex and EC memory foam), ML RMS was consistently associated with y-translation thresholds and, to a lesser extent, roll-tilt thresholds. Additionally, for these conditions, no consistent associations were noted between age-adjusted thresholds and MF or MV in either plane. In secondary conditions (EO firm, EC firm, EO Airex), age-adjusted thresholds for z-translation showed associations to MV and MF in both ML and AP planes.

#### 3.4.1 Age-adjusted thresholds and CoP RMS

**Figure 2:**
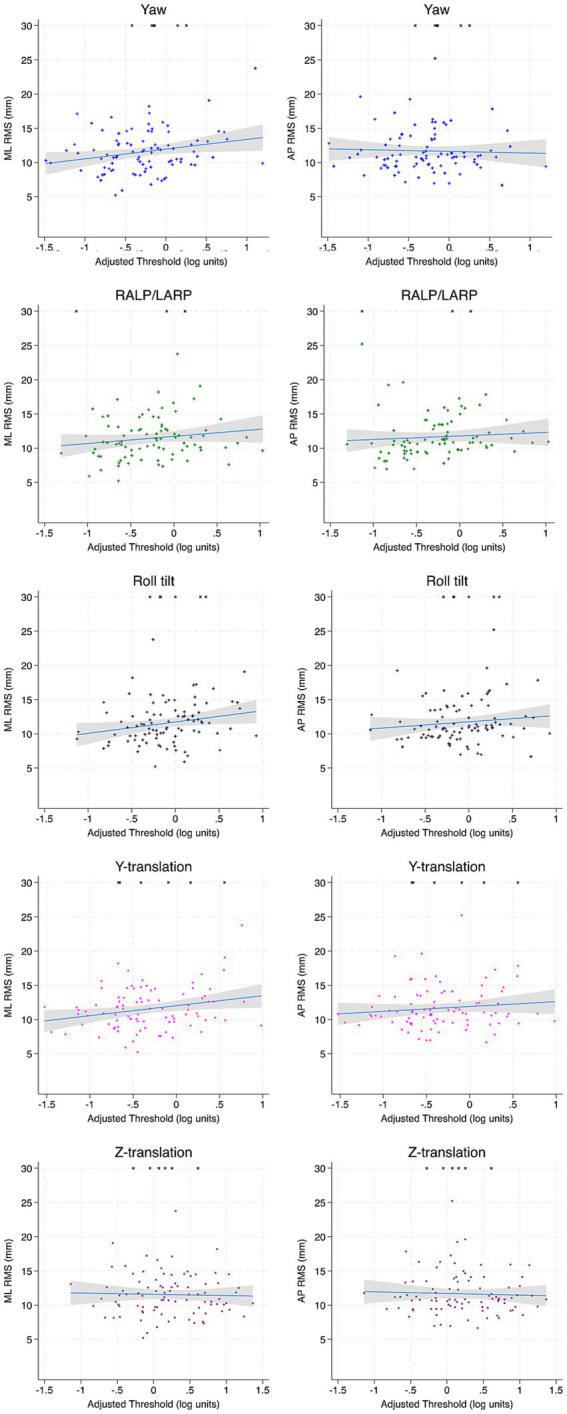
Scatter plots depicting the association between each age-adjusted vestibular threshold and ML and AP RMS of the CoP for condition 4 (EC Airex). Incomplete trials (not included in regression calculations) are depicted by arbitrarily assigning an RMS value of 30 mm. Linear fit is depicted in blue and the accompanying 95% confidence interval is depicted in gray. ML, mediolateral; AP, anterior–posterior; RMS, root mean square distance.

**Figure 3:**
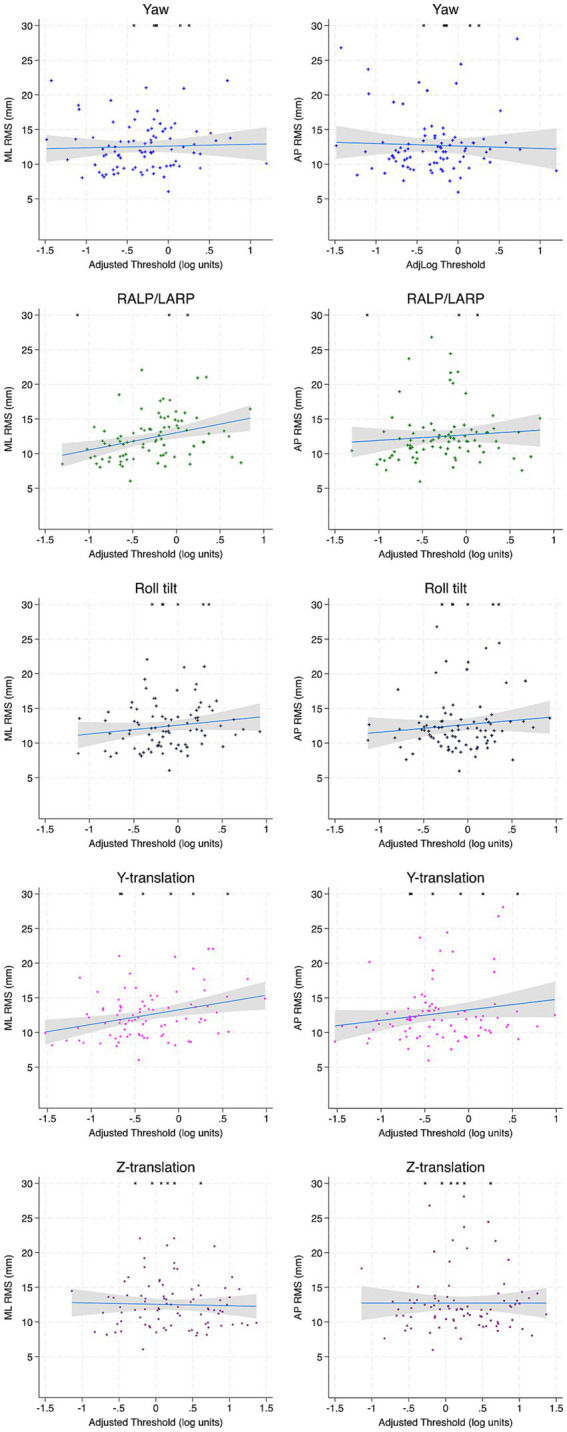
Scatter plots depicting the association between each age-adjusted vestibular threshold and ML and AP RMS of the CoP for condition 5 (EC memory foam). Incomplete trials (not included in regression calculations) are arbitrarily assigned an RMS value of 30 mm. Linear fit is depicted in blue and the accompanying 95% confidence interval is depicted in gray. ML, mediolateral; AP, anterior–posterior; RMS, root mean square distance.

In multivariable regression analyses, ML and AP RMS for Condition 4 (EC Airex) were not significantly related to any age-adjusted threshold (Table 4; Figure 2). For Condition 5 (EC memory foam), ML RMS showed a significant positive association with age-adjusted y-translation thresholds (*β* = 2.1, *p* = 0.005) and a significant negative association to yaw rotation thresholds (*β* = −1.58, *p* = 0.007) were identified (Table 4; Figure 3). No significant relationships between AP RMS and any threshold were identified in full regression analyses.

**Table 4:** Full multi-variable regression analyses assessing the relationship between each age-adjusted threshold to ML RMS and AP RMS during Conditions 4 and 5.

	Mediolateral (ML) RMS				Anterior–posterior (AP) RMS			
Threshold	*β*	SE	*t*	*p*-value	*β*	SE	*t*	*p*-value
Condition 4: eyes closed, Airex foam (*n* = 88)								
Yaw	0.63 (0.03)	0.87 (0.96)	0.72 (0.04)	0.475 (0.98)	1.66 **(−1.74)**	2.32 **(0.69)**	0.72 **(−2.51)**	0.475 (**0.014**)
RALP/LARP	−0.50 (−0.24)	0.64 (0.76)	−0.77 (−0.32)	0.444 (0.748)	−0.25 (1.27)	1.96 (0.76)	−0.13 (1.67)	0.898 (0.099)
Roll tilt	1.30 (1.40)	1.11 (1.21)	1.17 (1.14)	0.245 (0.257)	2.62 (1.34)	1.95 (1.08)	1.34 (1.24)	0.183 (0.218)
y-translation	1.36 (0.70)	0.90 (0.62)	1.50 (1.13)	0.137 (0.261)	−0.41 (0.31)	0.43 (0.62)	−0.94 (1.14)	0.351 (0.613)
z-translation	−0.34 (0.76)	0.39 (0.79)	−0.86 (0.96)	0.391 (0.341)	−0.99 (0.70)	1.36 (0.62)	−0.73 (1.14)	0.467 (0.259)
Intercept	9.41 (11.10)	0.91 (0.82)	10.34 (13.41)	<0.001 (<0.001)	10.12 (10.90)	1.09 (0.67)	9.30 (16.37)	<0.001 (<0.001)
Condition 5: eyes closed, memory foam (*n* = 84)								
Yaw	**−1.58 (−2.40)**	**0.57 (0.82)**	**−2.76 (−2.91)**	**0.007 (0.005)**	−1.35 **(−3.47)**	0.81 **(1.17)**	−1.68 **(−2.96)**	0.097 **(0.004)**
RALP/LARP	1.49 (1.53)	0.95 (0.82)	1.57 (1.88)	0.124 (0.064)	1.54 (0.44)	1.38 (0.85)	1.11 (0.52)	0.270 (0.607)
Roll tilt	1.28 (1.16)	0.91 (0.86)	1.40 (1.35)	0.166 (0.180)	0.75 **(2.82)**	1.09 **(1.06)**	0.69 **(2.65)**	0.490 **(0.010)**
y-translation	**2.07** (0.86)	**0.71** (0.71)	**2.93** (1.20)	**0.005** (0.235)	−0.29 (1.13)	0.46 (0.88)	−0.63 (1.28)	0.533 (0.205)
z-translation	−0.51 **(1.30)**	0.43 **(0.61)**	−1.19 **(2.12)**	0.237 **(0.037)**	−0.01 (0.74)	0.96 (0.67)	−0.01 (1.10)	0.994 (0.274)
Intercept	10.23 (11.17)	0.91 (0.70)	11.27 (15.98)	<0.001 (<0.001)	12.21 (11.37)	1.69 (0.84)	7.21 (13.58)	<0.001 (<0.001)

Results from non-adjusted thresholds are presented in parentheticals. Values which are statistically significant (i.e. *p* ≤ 0.05) are bolded.

In univariable regression analyses, ML RMS was positively associated to roll tilt thresholds (*β* = 1.803, *p* = 0.045) for EC Airex and with y-translation thresholds for EC Memory foam (*β* = 2.382, *p* = 0.020) similar to multivariable regression analyses. However, roll tilt thresholds failed to reach significance after correction for multiple comparisons (*β* = 1.844, *p* = 0.130). AP RMS remained unrelated to any age-adjusted threshold in both primary conditions (Table 5).

**Table 5:** Univariable regression analyses assessing the relationship between ML RMS and each age-adjusted threshold during Conditions 4 and 5.

	Mediolateral (ML) RMS				Anterior–posterior (AP) RMS			
Threshold	*β*	SE	*t*	*p*-value	*β*	SE	*t*	*p*-value
Condition 4: eyes closed, Airex foam (*n* = 93)								
Yaw	1.18 (1.36)	0.66 (0.63)	1.79 (2.17)	0.385 (0.331)	1.67 (0.04)	1.81 (0.53)	0.92 (0.07)	>0.99 (>0.99)
RALP/LARP*	0.64 (1.41)	0.46 (0.58)	1.39 (2.42)	0.845 (0.189)	0.58 (1.72)	0.57 (0.71)	1.00 (2.42)	>0.99 (0.170)
Roll tilt	**1.80 (2.15)**	**0.70 (0.56)**	**2.62 (3.80)**	**0.045 (0.010)**	0.58 (1.59)	0.75 (0.83)	0.77 (1.90)	>0.99 (0.610)
y-translation	1.84 (1.37)	1.01 (0.53)	1.82 (2.59)	0.360 (0.110)	2.95 (0.95)	2.34 (0.53)	1.26 (1.79)	>0.99 (0.771)
z-translation	−0.33 **(1.42)**	0.371 **(0.49)**	−0.89 **(2.91)**	>0.99 **(0.050)**	−0.29 (1.36)	0.34 (0.54)	−0.87 (2.49)	>0.99 (0.149)
Condition 5: eyes closed, memory foam (*n* = 87)								
Yaw	0.21 (0.39)	0.51 (0.78)	0.41 (0.50)	>0.99 (>0.99)	−0.11 (−0.15)	0.71 (1.14)	−0.15 (−0.14)	>0.99 (>0.99)
RALP/LARP**	1.26 **(2.32)**	0.77 **(0.60)**	1.62 **(3.87)**	0.540 **(0.001)**	−0.26 (1.20)	0.66 (0.57)	−0.39 (2.08)	>0.99 (0.412)
Roll tilt	1.94 **(2.75)**	0.86 **(0.81)**	2.26 **(3.42)**	0.130 **(0.010)**	2.20 (3.01)	1.52 (1.27)	1.45 (2.38)	0.750 (0.200)
y-translation	**2.38 (2.08)**	**0.80 (0.53)**	**2.99 (3.94)**	**0.020** (**0.001**)	0.92 (1.54)	1.15 (0.73)	0.81 (2.09)	>0.99 (0.385)
z-translation	−0.34 **(2.42)**	0.40 **(0.49)**	−0.84 **(5.00)**	>0.99 **(<0.001)**	−0.25 (1.68)	0.45 (0.69)	−0.56 (2.55)	>0.99 (0.168)

Results from analyses assessing nonadjusted thresholds are presented in parentheticals. *p*-values represent corrected *p*-values using a Bonferroni adjustment applied across each plane (i.e., corrected p = p*10). *N = 87; **N = 84. Values which are statistically significant (i.e., corrected *p* ≤ 0.05) are bolded.

For secondary balance conditions (Conditions 1–3), y-translation age-adjusted thresholds were associated with ML RMS in full regression analyses for all conditions (Supplementary Table 4) and Condition 2 (*β* = 1.193, *p* = 0.010) and 3 (*β* = 1.463, *p* = 0.050) in uni-variable models (Supplementary Table 5). Additionally, AP RMS in Condition 3 was related to roll tilt (*β* = 1.54, *p* = 0.004) and yaw rotation (*β* = −0.84, *p* = 0.021) in full regression analyses and y-translation thresholds (*β* = 1.248, *p* = 0.025).

#### 3.4.2 Age-adjusted thresholds and mean velocity (MV)

**Figure 4:**
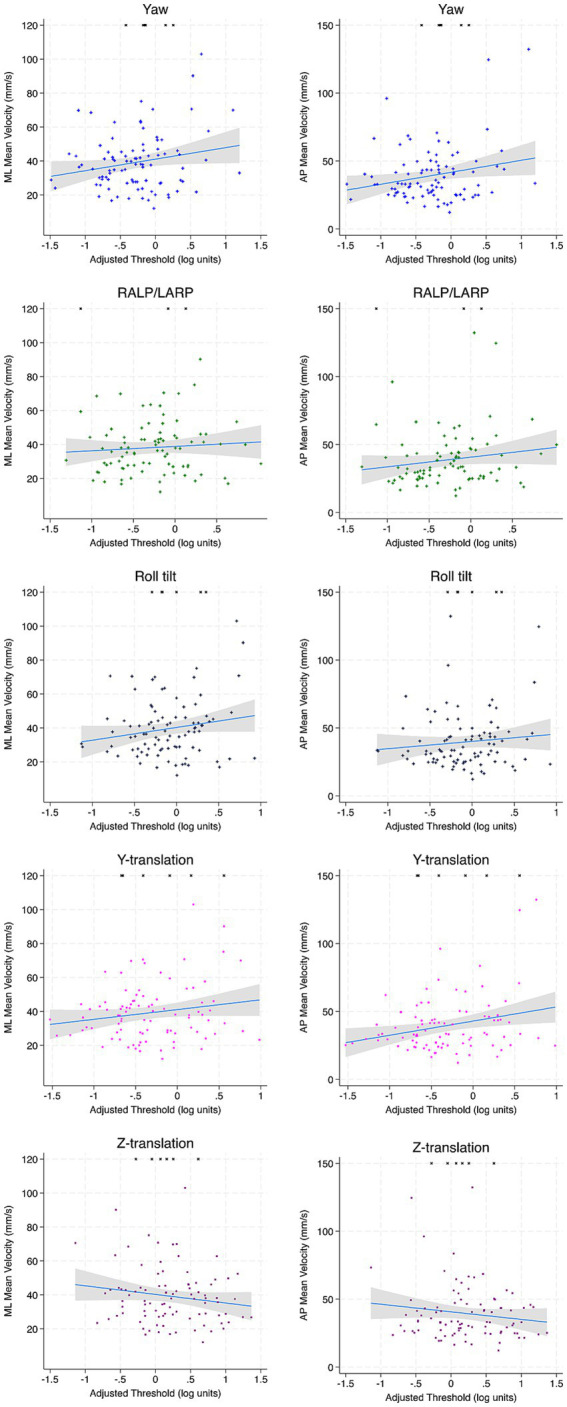
Scatter plots depicting the association between each vestibular threshold and ML and AP MV of the CoP for condition 4 (EC Airex). Incomplete trials (not included in regression calculations) are depicted by arbitrarily assigning an RMS value of 120 mm. Linear fit is depicted in blue and the accompanying 95% confidence interval is depicted in gray. ML, mediolateral; AP, anterior–posterior; MV, mean velocity.

**Figure 5:**
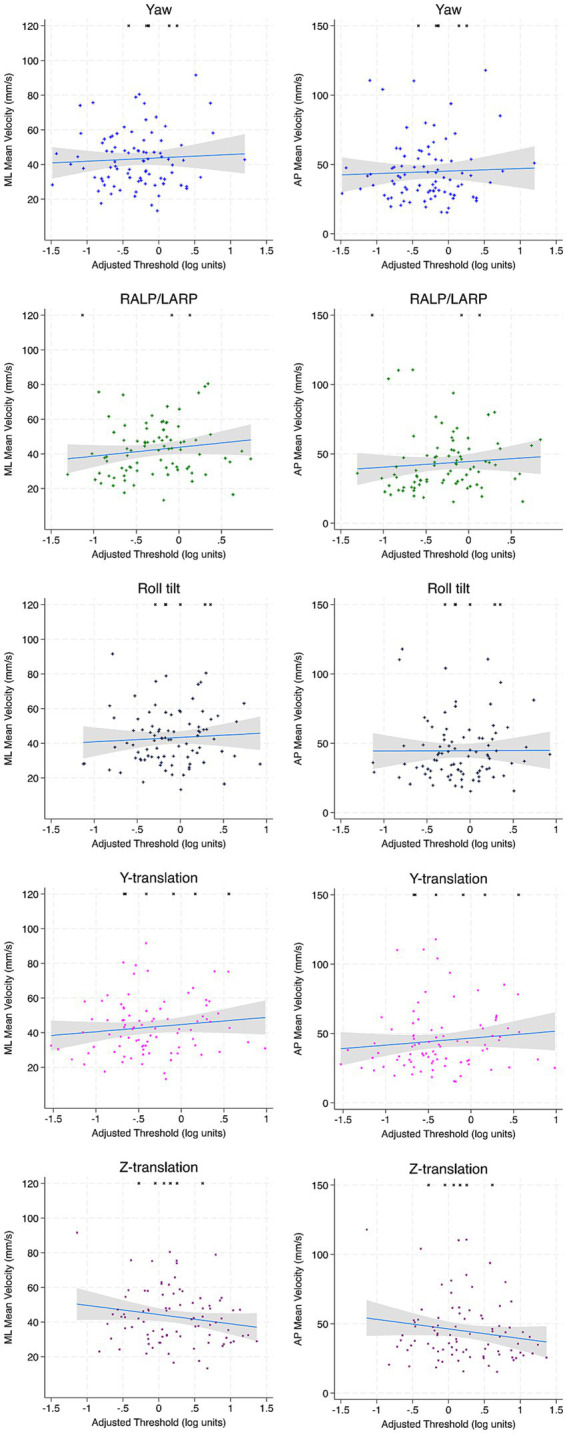
Scatter plots depicting the association between each vestibular threshold and ML and AP MV of the CoP for condition 5 (EC memory foam). Incomplete trials (not included in regression calculations) are depicted by arbitrarily assigning an RMS value of 150 mm/s. Linear fit is depicted in blue and the accompanying 95% confidence interval is depicted in gray. ML, mediolateral; AP, anterior–posterior; MV, mean velocity.

For Condition 4 and Condition 5, no significant associations between ML and AP MV were noted to any age-adjusted threshold motion in full regression analyses or univariable regression analyses (Tables 6, 7; Figures 4, 5).

**Table 6:** Full multi-variable regression analyses assessing the relationship between each age-adjusted threshold to ML MV and AP MV during Conditions 4 and 5.

	ML mean velocity				AP mean velocity			
Threshold	*β*	SE	*t*	*p*-value	*β*	SE	*t*	*p*-value
Condition 4: eyes closed, Airex foam (*n* = 88)								
Yaw	1.77 (−0.49)	4.34 (3.98)	0.40 (−0.12)	0.688 (0.902)	5.63 (1.35)	8.95 (7.02)	0.62 (0.19)	0.531 (0.848)
RALP/LARP	−1.05 (3.30)	3.31 (4.24)	−0.32 (0.78)	0.752 (0.438)	3.31 (7.22)	4.14 (5.21)	0.80 (1.39)	0.426 (0.169)
Roll tilt	4.30 (4.46)	6.02 (5.82)	0.71 (0.77)	0.477 (0.446)	1.28 (0.74)	8.78 (8.72)	0.14 (0.09)	0.885 (0.932)
y-translation	4.33 (2.78)	4.78 (4.09)	0.91 (0.68)	0.368 (0.446)	9.24 (3.72)	6.37 (4.45)	1.45 (0.84)	0.151 (0.405)
z-translation	−3.33 (2.05)	2.15 (4.01)	−1.55 (0.51)	0.125 (0.610)	−4.23 (3.72)	2.66 (4.45)	−1.59 (0.76)	0.115 (0.451)
Intercept	34.31 (38.20)	5.59 (4.64)	6.14 (8.24)	<0.001 (<0.001)	28.21 (39.00)	7.88 (5.47)	3.58 (7.12)	<0.001 (<0.001)
Condition 5: eyes closed, memory foam (*n* = 84)								
Yaw	−4.82 **(−8.18)**	3.26 **(3.32)**	−1.48 **(−2.46)**	0.143 **(0.016)**	−5.82 (−8.92)	4.99 (5.77)	−1.17 (−1.55)	0.248 (0.126)
RALP/LARP	2.38 (4.61)	4.86 (3.87)	0.49 (1.19)	0.625 (0.237)	3.77 (5.63)	5.81 (5.45)	0.65 (1.03)	0.518 (0.304)
Roll tilt	4.15 (5.78)	5.12 (4.97)	0.81 (1.16)	0.420 (0.249)	3.52 (4.63)	6.57 (7.98)	0.54 (0.58)	0.594 (0.563)
y-translation	3.84 (1.75)	4.04 (3.59)	0.95 (0.49)	0.345 (0.626)	3.46 (0.96)	4.96 (4.32)	0.70 (0.33)	0.487 (0.825)
z-translation	−3.51 **(6.28)**	1.84 **(2.62)**	−1.93 **(2.39)**	0.058 **(0.019)**	−3.97 **(9.09)**	2.17 **(3.74)**	−1.83 **(2.43)**	0.071 **(0.017)**
Intercept	42.10 (36.77)	5.48 (3.35)	7.68 (10.92)	<0.001 (<0.001)	44.28 (35.33)	8.33 (4.72)	5.31 (7.45)	<0.001 (<0.001)

Results from analyses assessing non-adjusted thresholds are presented in parentheticals. Values which are statistically significant (i.e. *p* ≤ 0.05) are bolded.

**Table 7:** Univariable regression analyses assessing the relationship between ML MV and AP MV and each age-adjusted threshold during Conditions 4 and 5.

	ML mean velocity				AP mean velocity			
Threshold	*β*	SE	*t*	*p*-value	*β*	SE	*t*	*p*-value
Condition 4: eyes closed, Airex foam (*n* = 93)								
Yaw	7.81 (9.00)	4.39 (3.64)	1.79 (2.47)	0.777 (0.160)	11.75 (10.52)	7.50 (5.19)	1.57 (2.03)	>0.99 (0.451)
RALP/LARP*	1.39 (8.43)	3.31 (3.21)	0.39 (2.63)	>0.99 (0.100)	9.85 **(13.26)**	3.58 **(3.93)**	2.75 **(3.37)**	0.073 **(0.014)**
Roll tilt	8.82 (11.03)	6.02 (4.58)	1.36 (2.41)	>0.99 (0.181)	7.03 (10.81)	7.55 (4.81)	0.93 (2.25)	>0.99 (0.271)
y-translation	5.95 (7.87)	4.78 (3.19)	1.26 (2.47)	>0.99 (0.166)	12.35 (9.72)	7.71 (3.88)	1.60 (2.51)	>0.99 (0.138)
z-translation	−3.34 (7.32)	2.15 (2.87)	−1.55 (2.55)	0.839 (0.133)	−4.05 **(8.64)**	2.27 **(2.53)**	−1.78 **(3.42)**	0.787 **(0.010)**
Condition 5: eyes closed, memory foam (*n* = 87)								
Yaw	−4.82 (3.47)	3.26 (3.41)	−1.48 (1.02)	0.713 (>0.99)	3.69 (4.79)	5.29 (5.10)	0.70 (0.94)	>0.99 (>0.99)
RALP/LARP**	3.18 **(8.83)**	4.86 **(2.77)**	0.49 **(3.19)**	>0.99 **(0.020)**	7.41 (10.44)	4.08 (3.85)	1.81 (2.71)	0.732 (0.084)
Roll tilt	4.15 (9.87)	5.12 (3.66)	0.81 (2.70)	>0.99 (0.088)	1.74 (10.78)	6.32 (5.76)	0.28 (1.87)	>0.99 (0.655)
y-translation	3.84 (6.04)	4.04 (2.65)	0.95 (2.28)	>0.99 (0.253)	3.44 (7.13)	5.15 (3.25)	0.67 (2.19)	>0.99 (0.311)
z-translation	−3.51 **(8.60)**	1.83 **(2.20)**	−1.93 **(3.90)**	0.290 **(<0.001)**	−5.24 **(11.01)**	2.52 **(3.22)**	−2.09 **(3.42)**	0.395 **(0.011)**

Results from analyses assessing non-adjusted thresholds are presented in parentheticals. *p*-values represent corrected *p*-values using a Bonferroni adjustment applied across each plane (i.e., corrected p = p*10). *N = 87; **N = 84. Values which are statistically significant (i.e., corrected *p* ≤ 0.05) are bolded.

In contrast, for the secondary conditions (Conditions 1–3), multivariable models revealed a consistent pattern of negative associations between age-adjusted z-translation thresholds and MV in both planes (Supplementary Tables 10, 11), excluding Condition 1 & 2 for AP MV. A significant association between y-translation was noted to ML MV for Condition 1 (*β* = 2.39, *p* = 0.029) and AP MV for Condition 3 (*β* = 5.87, *p* = 0.028).

#### 3.4.3 Age-adjusted thresholds and mean frequency (MF)

**Figure 6:**
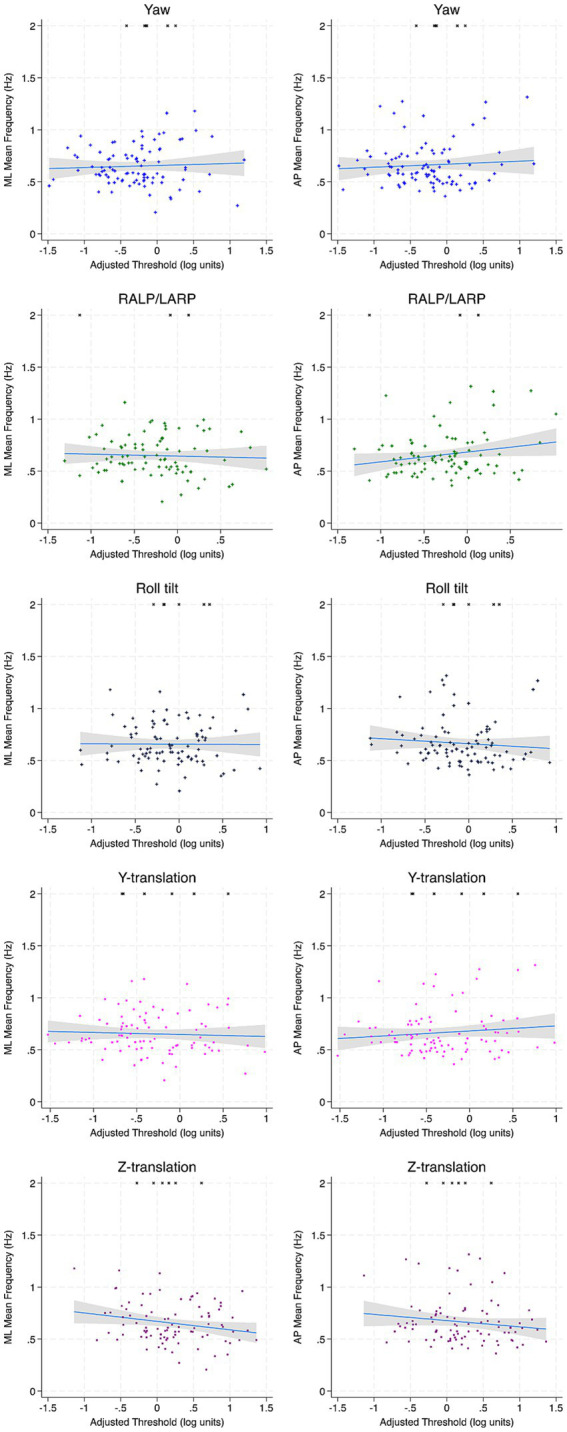
Scatter plots depicting the association between each vestibular threshold and ML and AP MF of the CoP for Condition 4 (EC Airex). Incomplete trials (not included in regression calculations) are depicted by arbitrarily assigning an RMS value of 2 Hz. Linear fit is depicted in blue and the accompanying 95% confidence interval is depicted in gray. ML, mediolateral; AP, anterior–posterior; MF, mean frequency.

**Figure 7:**
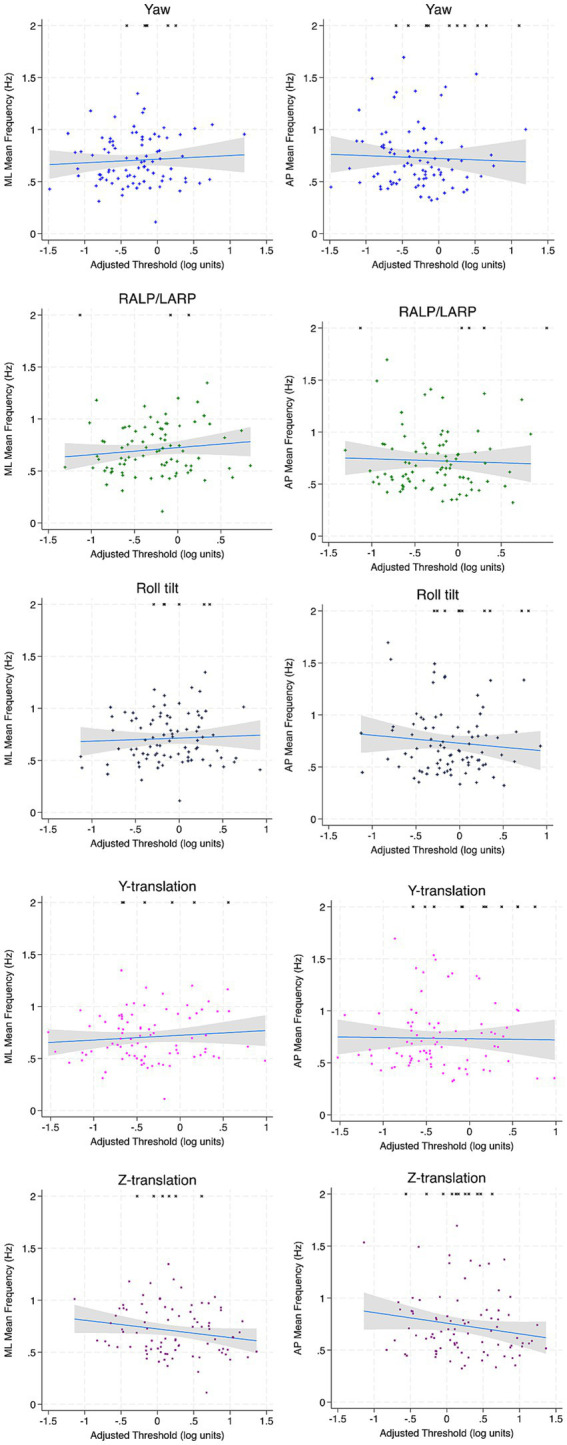
Scatter plots depicting the association between each vestibular threshold and ML and AP MF of the CoP for condition 5 (EC memory foam). Incomplete trials (not included in regression calculations) are depicted by arbitrarily assigning an RMS value of 2 Hz. Linear fit is depicted in blue and the accompanying 95% confidence interval is depicted in gray. ML, mediolateral; AP, anterior–posterior; MF, mean frequency.

In both multivariable and univariable models for Conditions 4 and 5, no significant associations were identified between ML or AP MF and any age-adjusted threshold, except for a single negative association with age-adjusted z-translation thresholds in Condition 5 (*β* = −0.060, *p* = 0.037) in multivariable analyses (Tables 8, 9; Figures 6, 7).

**Table 8:** Full regression analyses assessing the relationship between each age-adjusted threshold to ML RMS and AP MF during Conditions 4 and 5.

	ML mean frequency				AP mean frequency			
Threshold	*β*	SE	*t*	*p*-value	*β*	SE	*t*	*p*-value
Condition 4: eyes closed, Airex foam (*n* = 88)								
Yaw	0.006 (0.025)	0.054 (0.055)	0.11 (0.46)	0.915 (0.646)	0.023 (0.002)	0.067 (0.064)	0.34 (0.02)	0.733 (0.981)
RALP/LARP	−0.019 (0.011)	0.044 (0.055)	−0.43 (0.21)	0.670 (0.835)	0.125 (0.138)	0.075 (0.071)	1.53 (1.92)	0.131 (0.058)
Roll tilt	0.006 (0.033)	0.064 (0.060)	0.09 (0.56)	0.929 (0.580)	−0.106 (−0.110)	0.078 (0.076)	−1.36 (−1.44)	0.178 (0.155)
y-translation	−0.022 (−0.01)	0.057 (0.048)	−0.47 (−0.24)	0.643 (0.812)	0.045 (−0.009)	0.057 (0.047)	0.79 (−0.20)	0.430 (0.842)
z-translation	−0.027 (−0.011)	0.026 (0.043)	−1.04 (−0.12)	0.303 (0.902)	−0.035 (0.036)	0.026 (0.050)	−1.37 (0.483)	0.175 (0.842)
Intercept	0.709 (0.666)	0.07 (0.051)	10.19 (12.83)	<0.001 (<0.001)	0.654 (0.652)	0.082 (0.057)	8.00 (11.28)	<0.001 (<0.001)
Condition 5: eyes closed, memory foam (*n* = 84)								
Yaw	0.027 (−0.013)	0.059 (0.063)	0.54 (−0.21)	0.591 (0.834)	−0.034 (−0.052)	0.085 (0.096)	−0.40 (−0.55)	0.694 (0.586)
RALP/LARP	0.060 (0.060)	0.073 (0.063)	0.82 (0.96)	0.413 (0.342)	0.044 (0.080)	0.103 (0.102)	0.43 (0.79)	0.671 (0.433)
Roll tilt	−0.043 (0.080)	0.074 (0.079)	−0.59 (0.11)	0.558 (0.914)	−0.076 (−0.04)	0.095 (0.112)	−0.71 (−0.36)	0.482 (0.717)
y-translation	0.050 (0.017)	0.071 (0.054)	0.71 (0.33)	0.483 (0.745)	−0.018 (−0.022)	0.082 (0.075)	−0.22 (−0.29)	0.827 (0.775)
z-translation	−0.060 (0.042)	0.028 (0.041)	−2.13 (1.02)	**0.037 (0.312)**	−0.048 (0.054)	0.031 (0.063)	−1.56 (0.85)	0.124 (0.397)
Intercept	0.722 (0.691)	0.083 (0.049)	8.68 (13.95)	<0.001 (<0.001)	0.861 (0.679)	0.115 (0.075)	7.47 (8.94)	<0.001 (<0.001)

Results from analyses using non-adjusted thresholds are presented in parentheticals. Values which are statistically significant (i.e. *p* ≤ 0.05) are bolded.

**Table 9:** Univariable regression analyses assessing the relationship between ML MF and AP MF and each age-adjusted threshold during Conditions 4 and 5.

	ML mean frequency				AP mean frequency			
Threshold	*β*	SE	*t*	*p*-value	*β*	SE	*t*	*p*-value
Condition 4: eyes closed, Airex foam (*n* = 93)								
Yaw	0.099 (0.046)	0.106 (0.049)	0.94 (1.75)	>0.99 (>0.99)	0.061 (0.049)	0.058 (0.047)	1.05 (1.04)	>0.99 (>0.99)
RALP/LARP*	0.036 (0.054)	0.052 (0.036)	0.69 (1.50)	>0.99 (>0.99)	0.115 (0.100)	0.059 (0.044)	1.96 (2.26)	0.530 (0.267)
Roll tilt	0.141 (0.018)	0.146 (0.036)	0.96 (1.32)	0.99 (>0.99)	−0.029 (0.049)	0.071 (0.047)	−0.41 (0.85)	>0.99 (>0.99)
y-translation	0.009 (0.006)	0.063 (0.038)	0.14 (0.28)	0.99 (>0.99)	0.060 (0.050)	0.059 (0.040)	1.01 (1.25)	>0.99 (>0.99)
z-translation	−0.037 (0.001)	0.027 (0.023)	−1.39 (1.32)	>0.99 (>0.99)	−0.042 (0.061)	0.024 (0.033)	−1.77 (1.87)	0.801 (0.647)
Condition 5: eyes closed, memory foam (*n* = 87)								
Yaw	0.044 (0.049)	0.05 (0.100)	0.89 (1.04)	>0.99 (>0.99)	0.010 (0.016)	0.070 (0.075)	0.01 (0.22)	>0.99 (>0.99)
RALP/LARP**	0.075 (0.100)	0.043 (0.044)	1.75 (2.26)	0.834 (0.266)	0.003 (0.059)	0.075 (0.068)	1.07 (0.88)	>0.99 (>0.99)
Roll tilt	−0.003 (0.049)	0.069 (0.057)	−0.05 (0.85)	>0.99 (>0.99)	−0.049 (−0.008)	0.092 (0.076)	−0.53 (0.09)	>0.99 (>0.99)
y-translation	0.039 (0.050)	0.061 (0.040)	0.63 (1.25)	>0.99 (>0.99)	−0.045 (0.004)	0.069 (0.048)	−0.64 (0.09)	>0.99 (>0.99)
z-translation	−0.059 (0.061)	0.025 (0.033)	−2.39 (1.87)	0.190 (0.648)	−0.071 (0.039)	0.034 (0.046)	−2.11 (0.85)	0.379 (>0.99)

Results from analyses assessing non-adjusted thresholds are presented in parentheticals. *p*-values represent corrected *p*-values using a Bonferroni adjustment applied across each plane (i.e., corrected p = p*10). *N = 87; **N = 84. Values which are statistically significant (i.e., corrected *p* ≤ 0.05) are bolded.

For the secondary conditions (Conditions 1–3), age-adjusted z-translation thresholds were associated to ML MF for Condition 2 (*β* = −0.068, *p* = 0.009) and Condition 3 (*β* = −0.015, *p* = 0.044) and AP MF for Condition 3 (*β* = 0.072, *p* = 0.001) in multivariable analyses (Supplementary Table 12). AP MF was also associated to z-translation thresholds for Condition 1 (*β* = –0.075, *p* = 0.005) and Condition 3 (*β* = −0.076, *p* < 0.001) in univariable analyses (Supplementary Table 13).

### 3.5 Relationships between non-adjusted thresholds and CoP

We also analyzed relationships to non-adjusted thresholds (i.e., thresholds in the log domain) to distinguish effects attributable to vestibular sensory noise beyond chronological age from those reflecting the combined influence of age and vestibular decline. In general, when using non-adjusted thresholds, more associations were identified, particularly for z-translation.

#### 3.5.1 Thresholds and CoP RMS

In multi-variable analyses, non-adjusted yaw rotation thresholds showed a significant negative association with AP RMS in the primary vestibular conditions (Table 4; Figure 2). For Condition 5 (EC Memory foam), yaw rotation thresholds were negatively associated with both ML RMS (*β* = −2.40, *p* = 0.005) and AP RMS (*β* = −3.47, *p* = 0.007). ML RMS was positively associated with z-translation thresholds (*β* = 1.30, *p* = 0.037) and AP RMS was positively associated with roll-tilt thresholds (*β* = 2.82, *p* = 0.010).

In univariable analyses, ML RMS was positively associated with roll tilt thresholds for both Condition 4 (*β* = 2.15, *p* = 0.010) and Condition 5 (*β* = 2.75, *p* = 0.010; Table 5). As well, for Condition 5, a significant positive association between RALP/LARP tilt (*β* = 2.32, *p* = 0.001), y-translation (*β* = 2.38, *p* = 0.001) and z-translation thresholds (*β* = 2.42, *p* < 0.001) were seen. However, AP RMS was not associated with any threshold (Table 5).

For secondary conditions (Conditions 1–3), a significant association between ML RMS was seen for y-translation in multivariable regression analyses (Supplementary Table 8). In univariable analyses, y-translation was also positively associated ML RMS in Condition 2 (*β* = 1.25, *p* < 0.001) and 3 (*β* = 1.51, *p* < 0.001) and AP RMS in Condition 3 (*β* = 0.91, *p* = 0.030; Supplementary Table 9). In univariable regression analyses, a significant association was seen between z-translation to ML RMS for Condition 1–3 and to AP RMS for Condition 2 (*β* = 0.87, *p* = 0.045). Negative associations were noted between RALP/LARP tilt to ML RMS in Condition 1 (*β* = −0.82, *p* = 0.032) and yaw rotation to AP RMS in Condition 3 (*β* = −0.85, *p* = 0.021).

#### 3.5.2 Thresholds and mean velocity (MV)

In multivariable analyses for our primary vestibular conditions, no consistent pattern emerged between non-adjusted thresholds and MV (Table 6). In Condition 5, yaw thresholds were negatively associated to ML MV (*β* = −8.88, *p* = 0.016) and z-translation thresholds were positively associated to ML MV (*β* = 6.26, *p* = 0.019) and AP MV (*β* = 9.09, *p* = 0.0017). Univariable regression analyses revealed negative associations between z-translation to ML MV for Condition 5 (*β* = 8.60, *p* = 0.001) and AP MV for Condition 4 (*β* = −4.05, *p* = 0.010) and Condition 5 (*β* = −5.24, *p* = 0.010). RALP/LARP tilt thresholds were also associated with ML MV in Condition 5 (*β* = 8.38, *p* = 0.020) and AP MV in Condition 4 (*β* = 13.20, *p* = 0.014; Table 7).

For secondary conditions (Condition 1–3), z-translation thresholds were associated to ML MV for Condition 1 (*β* = 1.41, *p* = 0.030) and AP MV for Condition 2 in full regression analyses (*β* = 4.51, *p* = 0.009; Supplementary Table 8) and both ML MV and AP MV in univariable analyses (Supplementary Table 9). Y-translation thresholds were also associated with AP MV for Condition 1in multivariable analyses (*β* = 1.10, *p* = 0.043) and univariable analyses (*β* = 2.99, *p* = 0.0131), and Condition 3 in multivariable analyses (*β* = 4.37, *p* = 0.038). Additionally, roll tilt thresholds were positively related to ML MV for Condition 2 (*β* = 6.13, *p* = 0.018) and Condition 3 (*β* = 6.15, *p* = 0.013).

#### 3.5.3 Thresholds and mean frequency (MF)

In both multivariable and univariable analyses for our primary conditions (Condition 4 & 5), no significant associations with MF were identified to any vestibular threshold (Tables 8, 9).

For analyses assessing secondary conditions, only a significant association z-translation thresholds to ML MF for Condition 1 (*β* = 0.106, *p* = 0.002; Supplementary Table 12) was identified in multivariable analyses. No significant associations between any threshold to ML MF or AP MF were identified in univariable regression analyses (Supplementary Table 13).

## 4 Discussion

Across motions, we observed age-related threshold increases, but support for a two-segment model was not uniform across motions. The clearest evidence for a two-segment piecewise pattern occurred for y-translation, where model diagnostics showed a substantial statistically significant improvement over a linear fit. In contrast, yaw rotation, RALP/LARP tilt, and z-translation showed statistically significant improvement in nested model testing but only small improvements in information criteria, and roll tilt showed no meaningful advantage for a two-segment model. The exact age cutoffs and slopes varied across motion conditions, potentially reflecting different end-organ contributions, the general pattern of late-onset decline aligns with prior work despite absolute differences likely driven by sample and paradigm differences [e.g., (13)].

Age-adjusted thresholds were moderately interrelated, yet only y-translation, and to a lesser extent roll tilt, showed robust associations with quiet-stance sway. Given the number of comparisons across motions, balance metrics, planes, and conditions, we focus interpretation on *a priori* primary hypotheses (ML RMS in vestibular-dependent eyes-closed foam conditions). In our primary vestibular-dependent conditions, higher y-translation thresholds were consistently linked to greater ML RMS, whereas AP RMS showed no meaningful relationships. In contrast, MV and MF were largely unrelated to age-adjusted thresholds in the primary vestibular conditions. Analyses using non-adjusted thresholds yielded broadly similar patterns, suggesting that the association between y-translation thresholds and ML sway does not simply reflect parallel age-related changes, but instead supports a link between vestibular precision and sway variability.

### 4.1 Linear vs. two-segment fits

Age-related changes in vestibular perceptual thresholds were often consistent with a two-segment (piecewise) pattern; however, support for this model was motion-specific The strongest evidence for a two-segment pattern was observed for y-translation, where piecewise modeling yielded a ~ 15-point reduction in BIC relative to the linear model, suggesting that a large proportion of variance of each vestibular threshold was unexplained by age. In contrast, for several other motions the likelihood-ratio test favored the piecewise model but improvements in information criteria were small (ΔBIC < 1), suggesting that any advantage of the two-segment formulation for these motions may be statistically detectable but not practically meaningful. Consistent with this, 0.5 Hz roll tilt showed no advantage for a two-segment model.

Across motions, adjusted R^2^ values ranged from 0.15 to 0.51, indicating that age explained only a portion of the variance in thresholds and that substantial inter-individual differences in perceptual precision remained. These individual differences may reflect variability in sensory processing, but thresholds can also be influenced by non-sensory factors (e.g., attention, decision criteria).

In general, slopes were steeper for two-segment fits relative to linear slopes, suggesting that simple linear modeling may over-estimate age-related decline in young adults. Yaw rotation, for example, had one of the highest age cutoffs and relatively low adjusted R^2^, suggesting individual differences not captured by age alone. In contrast, both 0.5 Hz roll tilt and 1 Hz z-translation displayed similar slopes between two-segment functions and linear fits, which is consistent with the lack of clear two-segment advantage for roll tilt and the low age cutoff for z-translation, where baseline and post-cutoff data span a similar age range.

### 4.2 Motion differences

Overall, the relationships between thresholds to each other are similar for our current study in comparison to other datasets. All unadjusted thresholds displayed significant moderate correlations suggesting that all thresholds captured differences in perceptual precision which may represent an individual trait. For age-adjusted thresholds, weak to moderate (r^2^ = 0.245–0.432) correlations were identified (Figures 2–7) suggesting substantial motion-specific variability in perception not explained by aging alone.

Our findings also indicate broadly similar perceptual precision and age effects between motions dominated by horizontal versus vertical canal inputs. At 2 Hz, yaw rotation (predominantly horizontal canals) and RALP/LARP tilt (predominantly vertical canals) thresholds did not differ significantly in magnitude [*t*(88) = −1.23, *p* = 0.216], consistent with prior work showing comparable yaw, roll, and pitch thresholds across frequencies (54). However, the age-cutoffs differed as yaw thresholds were stable until ~52 years of age and RALP/LARP tilt thresholds began to worsen at ~42 years of age. Prior data suggests that 2 Hz RALP/LARP tilt thresholds are largely canal driven (33) and undergo relatively limited central processing, whereas yaw rotation engages distinct central mechanisms, including velocity storage with longer time constants and smaller otolith contributions (55–58). Such differences in central processing may explain the later age of onset of age-related changes for yaw rotation.

Unique central processing of motion cues may also contributed to 0.5 Hz roll tilt, which relies on integration of canal and otolith cues (33, 40) and was the only motion for which a two-segment model did not significantly outperform a linear fit. Notably, roll tilt thresholds also had one of the youngest apparent cutoffs (38 years of age), which may have limited the statistical advantage of the two-segment model.

For motions with predominant otolith contributions, at baseline prior to identified age-related changes, thresholds for z-translation were ~2-3x higher than baseline y-translation thresholds in line with past reports (13, 35, 36, 41). In the two-segment models, the age cutoff for y-translation (54 years) was later than z-translation thresholds (36 years); however, the slopes per decade after this age-cutoff were similar (i.e., 0.373 cm/s for y-translation, 0.312 for z-translation). Our earlier work suggests that earth-vertical (i.e., parallel to gravity) translations maximized vestibular contributions to perceptual sensitivity relative to earth-horizontal translations (i.e., perpendicular to gravity) (35, 41). Thus, the similar slopes despite different cutoffs raise the possibility that participants may have been able to compensate for age-related changes during y-translation using non-vestibular cues (e.g., tactile cues), but these compensatory strategies became less effective as age-related vestibular changes accumulated.

### 4.3 Comparisons of age-fits to past studies

Bermúdez-Rey et al. (13) first proposed that vestibular perceptual thresholds follow a two-segment age pattern, and our modeling, which deliberately mirrored their approach, generally supports this view. Both datasets show relatively low thresholds through young and middle adulthood followed by age-related increases, although our estimated cutoffs (≈37–54 years) span a slightly broader range than the ~40-year cutoff reported previously. These differences likely reflect a combination of distinct motion paradigms and sampling, particularly our intentional enrichment of older adults (60–84 years), which may have sharpened estimates of late-life changes while leaving fewer participants around the previously reported cutoff. Further, the current data was not equally distributed per decade. Sampling of participants in their 30’s clustered to the early 30’s with only three participants over 35 years and similarly only 5 of the 14 participants in their 40’s were under 45. Thus, we did not sample a large number of subjects around the previously identified age-cutoff of 42 years and may have yielded fits that identified the age-cutoff on either side of where age-related changes begin.

For 1 Hz y- and z-translation, which were assessed in both studies, baseline thresholds were similar, but we observed a later age cutoff and slightly steeper slope for y-translation and an earlier cutoff and shallower slope for z-translation compared with Bermúdez-Rey et al. (13). Given that geometric means per decade were similar across cohorts, these differences likely reflect how the two-segment models partitioned the age range rather than fundamentally different aging effects, and both studies converge on a larger overall impact of age on z-translation than on y-translation (13, 37, 59). Notably, our expanded upper age range also allowed us to detect a statistically significant age effect for yaw rotation, which may have been missed in prior work that included relatively few adults over 70 years (13, 37, 60, 61). In our data, yaw rotation threshold had one of the highest age cutoffs at 52 years of age, and increases with age were most pronounced in adults in 70’s and 80’s. Thus, without inclusion of a large enough number of adults over 70, it may not be possible to identify a significant age-effect.

### 4.4 Associations between vestibular thresholds and CoP metrics

Vestibular perceptual thresholds have been proposed to serve as an assay of neural noise (12, 21, 22) and models of balance control attribute spontaneous sway due to internal noise (19, 23–27). Thus, within this framework, a theoretical increase in sensory noise (i.e., higher vestibular thresholds) would be expected to yield an increase in spontaneous postural sway (see (62) for a review). However, multiple non-sensory factors (e.g., attention, decision criteria) may influence psychophysical measures and multiple motor factors (e.g., strength) may influence balance performance. However, consistent with this hypothesis, our primary analyses in vestibular-dependent conditions (eyes closed on Airex or memory foam) showed that higher y-translation thresholds, and to a lesser extent roll-tilt thresholds, were associated with greater ML RMS of the CoP for both age-adjusted and non-adjusted thresholds. Similar but weaker associations between y-translation thresholds and ML RMS were present under conditions with stronger visual and proprioceptive contributions (EO firm, EC firm, EO Airex), and the strength of association increased as vestibular contributions to balance became more prominent.

Using non-adjusted thresholds revealed a larger number of significant relationships, particularly for z-translation, which showed consistent associations with CoP metrics. Given that z-translation exhibited the largest age-related changes overall, these additional relationships likely reflect global age effects on both vestibular perception and postural control and underscore the importance of age adjustment when relating vestibular “noise” to sway. Despite theoretical expectations that both RMS and MV should reflect a common noise source (23), robust and consistent associations primarily between vestibular thresholds and ML RMS in vestibular-dependent conditions were identified, whereas MV and MF showed limited and less consistent relationships once age was accounted for. Positive associations between MV and several non-adjusted thresholds (e.g., z-translation, RALP/LARP tilt) again appeared to be driven predominantly by shared age effects rather than a specific vestibular noise mechanism.

Associations involving z-translation emerged primarily in secondary balance conditions (Conditions 1–3) and were atypical in direction, most often involving MV/MF rather than RMS, and were not reproduced consistently across conditions. In addition, the age-adjusted z-translation threshold showed weak interrelationships with other adjusted thresholds and the widest dispersion, suggesting substantial variability not explained by age alone. Although no individual observations were influential in any regression (DFBETAS below conventional thresholds), visual inspection suggested that a small subset of participants with high z-translation thresholds exhibited relatively low MV and MF across conditions, consistent with heterogeneous postural strategies.

Separately, a small number of isolated associations were counterintuitive (e.g., negative associations between yaw thresholds and RMS). Because these effects were intermittent across conditions and model specifications, we do not interpret them as evidence of a reliable physiological relationship. However, future research is needed to determine potential relationships between postural control strategies and vestibular thresholds. Overall, these findings complement prior work identifying relationships between sway variability during predominant vestibular contributions to roll tilt (13, 17, 18), y-translation (15), and z-translation (17). Our data generally aligns with these past findings as we were able to identify consistent associations between y-translation to ML RMS for both age-adjusted and non-adjusted thresholds. These findings suggest that y-translation thresholds may serve as a marker of vestibular contributions to mediolateral postural instability, which is associated with fall risk (14). Future research should explicitly examine if y-translation thresholds may serve as a marker of fall risk, which was not assessed in this current study.

We also extend our past work that assessed a subset of this data (*n* = 52) (17) by demonstrating these relationships across two levels of compliant surface (i.e., standard Airex, memory foam) in a larger sample. The higher failure rate and larger RMS on memory foam relative to Airex suggest that this condition more effectively down weights non-vestibular somatosensory cues and may therefore be particularly reliant upon vestibular contributions to maintain postural control.

### 4.5 Limitations

In our current sample, only a small number of subjects were unable to complete these conditions when standing on Airex (*n* = 6) and memory foam (*n* = 12) representing 6.25 and 12.3% of participants. Approximately 35% of adults were unable to complete similar condition in the NHANES dataset, which represents a nationally representative sample (4). This suggests that our sample represents an ultra-healthy older adult cohort, which likely reflects our strict inclusionary criteria. As non-completion occurred primarily in the vestibular-dependent eyes-closed foam conditions, our primary condition associations may be biased toward older adults with better balance capacity and may therefore underestimate relationships that would be observed in a more representative aging cohort.

The intent of our inclusion and exclusion criteria was to reduce the potential influence of medical conditions which may also impact vestibular function, thus, allowing us to focus on age-related vestibular contributions. However, while this allows us to potentially better isolate aging effects in the absence of other health conditions, the reported sample is not representative of the larger aging population in the United States. Future studies should examine more diverse samples in order to characterize the potential impact of co-occurring health conditions commonly found in aging adults (e.g., diabetes) and incorporate key covariates (e.g., anthropometrics, and relevant health conditions/medications) in modeling rather than relying solely on restrictive inclusion/exclusion criteria to address confounding health status.

Additionally, our balance trials were relatively long (67 s). Although this duration improves reliability of sway metric estimation, it may also introduce fatigue, attentional drift, or sensory adaptation, which could disproportionately affect older participants. Future work or analyses could explicitly evaluate time-varying sway within trials and compare shorter standardized epochs to determine the extent to which trial duration influences age effects and vestibular–balance associations.

An important limitation is that RALP/LARP thresholds could not be obtained for a subset of older participants because the required stimulus exceeded the platform maximum (2.5 deg./s). These observations could be considered censored, which can bias estimates if treated as missing not at random. Future studies designed to characterize high-threshold individuals would benefit from higher-capacity hardware or analytic approaches that explicitly model censoring (e.g., censored-regression/Tobit or survival-style methods), ideally paired with device ranges that reduce truncation in the oldest age groups.

## Data Availability

The raw data supporting the conclusions of this article will be made available by the authors, without undue reservation.
