# Supplementary material for: Two-segment aging of vestibular perceptual thresholds: motion-specific links to quiet-stance postural sway
Source: Front Neurol. 2026 Mar 6;17:1766090. doi: 10.3389/fneur.2026.1766090 (PMC13002458; doi:10.3389/fneur.2026.1766090)
Supplement: Supplementary file 1 [file Table_1.DOCX]

|  | **Yaw rotation** | **RALP/LARP tilt** | **Roll tilt** | **y-translation** | **z-translation** |
| --- | --- | --- | --- | --- | --- |
| **Log transformed thresholds** | | | | | |
| **Yaw rotation** | 1 |  |  |  |  |
| **RALP/LARP tilt** | 0.552  **(p<0.001)** | 1 |  |  |  |
| **Roll tilt** | 0.570  **(p<0.001)** | 0.623  **(p<0.001)** | 1 |  |  |
| **y-translation** | 0.525  **(p<0.001)** | 0.5949  **(p<0.001)** | 0.511  **(p<0.001)** | 1 |  |
| **z-translation** | 0.408  **(p<0.001)** | 0.631  **(p<0.001)** | 0.564  **(p<0.001***)* | 0.536  **(p<0.001)** | 1 |
| **Age-adjusted thresholds** | | | | | |
| **Yaw rotation** | 1 |  |  |  |  |
| **RALP/LARP tilt** | 0.267  **(p=0.012)** | 1 |  |  |  |
| **Roll tilt** | 0.310  **(p=0.002)** | 0.432  **(p<0.001)** | 1 |  |  |
| **y-translation** | 0.444  **(p<0.001)** | 0.262  **(p=0.014)** | 0.245  **(p=0.016)** | 1 |  |
| **z-translation** | -0.004  (p=0.978) | 0.056  (p=0.603) | 0.113  (p=0.280) | 0.095  (p=0.355) | 1 |

**Supplemental Table 1**. Pearson product correlation coefficients (i.e. Pearson’s r) examining the relationships between thresholds. Log transformed thresholds are displayed in the top half while age-adjusted thresholds are displayed in the bottom half. Statistically significant corrected p-values are bolded.

|  | **Condition 1: EO-Firm** | **Condition 2: EC-Firm** | **Condition 3:**  **EO-Airex** | **Condition 4:**  **EC-Airex** | **Condition 5:**  **EC-Memory** |
| --- | --- | --- | --- | --- | --- |
| 20-29 | 5.14 (1.57) | 5.51 (1.61) | 6.66 (1.22) | 10.54 (2.39) | 10.94 (2.60) |
| 30-39 | 4.96 (1.32) | 5.39 (1.12) | 7.04 (0.98) | 10.48 (2.57) | 11.06 (3.00) |
| 40-49 | 5.27 (1.62) | 6.00 (1.44) | 7.38 (1.46) | 11.14 (3.08) | 13.05 (3.79) |
| 50-59 | 3.91 (0.90) | 5.03 (0.99) | 6.59 (1.24) | 10.42 (1.40) | 12.54 (3.50) |
| 60-69 | 5.73 (2.08) | 6.09 (1.88) | 8.72 (2.50) | 14.52 (4.13) | 13.28 (2.22) |
| 70-79 | 6.00 (1.80) | 7.59 (3.11) | 9.87 (2.76) | 13.68 (2.13) | 16.12 (2.90) |
| 80-89 | 5.88 (2.00) | 7.67 (2.73) | 9.70 (1.63) | 11.56 (3.44) | 16.01 (3.09) |
| Total | 5.25 (1.69) | 6.04 (2.02) | 7.73 (2.07) | 11.58 (3.09) | 12.53 (3.40) |

**Supplemental Table 2**. Mean and standard deviation of ML CoP RMS for all participants who completed balance testing. EO= Eyes open, EC = Eyes closed

|  | **Condition 1: EO-Firm** | **Condition 2: EC-Firm** | **Condition 3:**  **EO-Airex** | **Condition 4:**  **EC-Airex** | **Condition 5:**  **EC-Memory** |
| --- | --- | --- | --- | --- | --- |
| 20-29 | 5.54 (2.10) | 5.99 (1.63) | 7.61 (2.08) | 10.72 (2.11) | 12.04 (4.20) |
| 30-39 | 5.81 (3.94) | 5.59 (1.64) | 7.93 (1.72) | 10.57 (2.53) | 13.18 (6.16) |
| 40-49 | 5.55 (1.23) | 6.20 (1.30) | 8.68 (2.02) | 11.30 (2.31) | 11.61 (2.98) |
| 50-59 | 4.26 (1.19) | 5.01 (1.03) | 7.34 (1.06) | 11.14 (1.56) | 11.91 (2.09) |
| 60-69 | 5.74 (2.21) | 6.58 (1.70) | 9.04 (3.02) | 16.37 (10.49) | 13.67 (3.64) |
| 70-79 | 5.52 (1.60) | 7.02 (1.94) | 9.11 (1.91) | 13.83 (2.72) | 15.84 (6.17) |
| 80-89 | 5.70 (1.88) | 8.06 (3.42) | 9.03 (1.31) | 12.82 (6.82) | 15.01 (3.79) |
| Total | 5.51 (2.37) | 6.23 (1.92) | 8.28 (2.08) | 12.08 (4.86) | 12.96 (4.62) |

**Supplemental Table 3.** Mean and standard deviations of AP CoP RMS for all participants who completed balance testing.

|  | **Condition 1: EO-Firm** | **Condition 2: EC-Firm** | **Condition 3:**  **EO-Airex** | **Condition 4:**  **EC-Airex** | **Condition 5:**  **EC-Memory** |
| --- | --- | --- | --- | --- | --- |
| 20-29 | 8.79 (2.44) | 12.07 (4.60) | 15.70 (3.84) | 34.10 (11.92) | 35.42 (11.25) |
| 30-39 | 8.37 (2.56) | 12.34 (3.78) | 16.45 (4.05) | 31.05 (10.68) | 35.88 (11.40) |
| 40-49 | 10.73 (3.99) | 14.48 (6.56) | 18.20 (4.20) | 35.09 (12.71) | 41.32 (16.41) |
| 50-59 | 10.48 (3.63) | 15.32 (5.95) | 21.11 (8.25) | 37.00 (12.87) | 46.37 (15.90) |
| 60-69 | 12.14 (4.45) | 20.00 (10.78) | 24.22 (5.49) | 49.36 (22.71) | 41.11 (24.35) |
| 70-79 | 17.97 (13.21) | 25.71 (12.81) | 28.82 (14.58) | 45.05 (20.62) | 44.03 (33.77) |
| 80-89 | 17.25 (8.39) | 24.83 (9.49) | 24.57 (17.66) | 40.57 (35.97) | 25.41 (28.96) |
| Total | 11.52 (6.86) | 16.76 (9.22) | 20.42 (9.43) | 37.95 (18.38) | 38.55 (20.45) |

**Supplemental Table 4.** Mean and standard deviation of ML Mean Velocity for each condition across age group.

|  | **Condition 1: EO-Firm** | **Condition 2: EC-Firm** | **Condition 3:**  **EO-Airex** | **Condition 4:**  **EC-Airex** | **Condition 5:**  **EC-Memory** |
| --- | --- | --- | --- | --- | --- |
| 20-29 | 8.01 (2.20) | 11.13 (4.30) | 13.99 (3.27) | 34.05 (12.63) | 36.36 (18.00) |
| 30-39 | 7.96 (2.84) | 11.20 (3.04) | 14.53 (4.41) | 28.62 (9.04) | 35.21 (19.24) |
| 40-49 | 8.63 (2.51) | 12.94 (5.81) | 15.08 (3.05) | 32.30 (10.43) | 37.69 (13.71) |
| 50-59 | 9.02 (2.72) | 14.06 (5.45) | 19.58 (6.25) | 39.91 (15.63) | 50.96 (18.47) |
| 60-69 | 12.39 (7.31) | 23.82 (19.17) | 26.48 (9.82) | 58.84 (39.44) | 46.36 (30.82) |
| 70-79 | 13.30 (4.89) | 22.34 (8.47) | 27.55 (13.42) | 45.94 (19.50) | 47.67 (41.52) |
| 80-89 | 21.20 (17.84) | 29.26 (16.62) | 22.93 (16.03) | 33.22 (25.74) | 27.82 (30.65) |
| Total | 10.59 (7.17) | 16.46 (11.41) | 19.13 (9.74) | 38.15 (21.68) | 40.02 (25.41) |

**Supplemental Table 5.** Mean and standard deviation of AP Mean Velocity for each condition across age group.

|  | **Condition 1: EO-Firm** | **Condition 2: EC-Firm** | **Condition 3:**  **EO-Airex** | **Condition 4:**  **EC-Airex** | **Condition 5:**  **EC-Memory** |
| --- | --- | --- | --- | --- | --- |
| **20-29** | 0.35 (0.14) | 0.43 (0.16) | 0.43 (0.14) | 0.64 (0.19) | 0.64 (0.21) |
| **30-39** | 0.34 (0.12) | 0.47 (0.14) | 0.43 (0.11) | 0.60 (0.16) | 0.60 (0.18) |
| **40-49** | 0.39 (0.13) | 0.48 (0.21) | 0.43 (0.10) | 0.62 (0.16) | 0.72 (0.25) |
| **50-59** | 0.51 (0.15) | 0.62 (0.23) | 0.58 (0.20) | 0.66 (0.19) | 0.79 (0.23) |
| **60-69** | 0.46 (0.21) | 0.59 (0.24) | 0.59 (0.19) | 0.76 (0.27) | 0.82 (0.23) |
| **70-79** | 0.64 (0.31) | 0.74 (0.32) | 0.69 (0.20) | 0.72 (0.22) | 0.86 (0.26) |
| **80-89** | 0.59 (0.18) | 0.65 (0.20) | 0.74 (0.21) | 1.00 (0.90) | 0.69 (0.20) |
| **Total** | 0.44 (0.21) | 0.55 (0.23) | 0.52 (0.19) | 0.68 (0.30) | 0.71 (0.23) |

**Supplemental Table 6.** Mean and standard deviation of ML MF for each condition across age group.

|  | **Condition 1: EO-Firm** | **Condition 2: EC-Firm** | **Condition 3:**  **EO-Airex** | **Condition 4:**  **EC-Airex** | **Condition 5:**  **EC-Memory** |
| --- | --- | --- | --- | --- | --- |
| **20-29** | 0.33 (0.11) | 0.42 (0.14) | 0.42 (0.08) | 0.64 (0.19) | 0.64 (0.21) |
| **30-39** | 0.33 (0.11) | 0.42 (0.10) | 0.42 (0.10) | 0.60 (0.16) | 0.60 (0.18) |
| **40-49** | 0.33 (0.07) | 0.43 (0.12) | 0.42 (0.09) | 0.62 (0.16) | 0.72 (0.25) |
| **50-59** | 0.46 (0.07) | 0.55 (0.15) | 0.59 (0.11) | 0.66 (0.19) | 0.79 (0.23) |
| **60-69** | 0.45 (0.21) | 0.75 (0.39) | 0.62 (0.18) | 0.76 (0.27) | 0.82 (0.23) |
| **70-79** | 0.46 (0.15) | 0.65 (0.28) | 0.61 (0.17) | 0.72 (0.22) | 0.86 (0.26) |
| **80-89** | 0.67 (0.36) | 0.78 (0.42) | 0.64 (0.08) | 1.00 (0.90) | 0.69 (0.20) |
| **Total** | 0.41 (0.18) | 0.54 (0.27) | 0.51 (0.15) | 0.68 (0.30) | 0.71 (0.23) |

**Supplemental Table 7.** Mean and standard deviation of AP MF for each condition across age group.

|  | **ML RMS** | | | | | **AP RMS** | | | |
| --- | --- | --- | --- | --- | --- | --- | --- | --- | --- |
|  | β | SE | t | p-value | β | | SE | t | p-value |
| **Condition 1:** Eyes Open, Firm Surface (n=89) | | | | | | | | | |
| Yaw | -0.26  (-0.52) | 0.34 (0.43) | -0.76  (-1.21) | 0.447 (0.229) | -0.03 (0.22) | | 0.41 (0.58) | -0.08 (0.37) | 0.935 (0.712) |
| RALP/LARP* | -0.43  **(-0.82)** | 0.36 **(0.37)** | -1.21  **(-2.18)** | 0.229 (**0.032**) | 0.77  (-0.35) | | 0.74 (0.58) | 1.04  (-0.61) | 0.321 (0.544) |
| Roll tilt | 0.67 (0.92) | 0.60 (0.52) | 1.12 (1.75) | 0.265 (0.083) | 0.22 (0.81) | | 0.62 (0.92) | 0.35 (0.88) | 0.726 (0.383) |
| y-translation | **0.81 (0.74)** | **0.31 (0.26)** | **2.64 (2.79)** | **0.010 (0.006)** | -0.17  (-0.02) | | 0.26 (0.57) | -0.67  (-0.03) | 0.506 (0.973) |
| z-translation | 0.21 (0.48) | 0.22 (0.27) | 0.96 (1.78) | 0.34 (0.078) | -0.55  (-0.50) | | 0.48 (0.38) | -1.13  (-2.33) | 0.26 (0.187) |
| Intercept | 4.12 (4.69) | 0.51 (0.30) | 8.10 (15.72) | <0.001  (<0.001) | 5.25 (5.88) | | 0.86 (0.45) | 6.11 (12.96) | <0.001  (<0.001) |
| **Condition 2:** Eyes Closed, Firm Surface (n=89) | | | | | | | | | |
| Yaw | -0.15  (-0.58) | 0.33 (0.49) | -0.45  (-1.17) | 0.656 (0.244) | -0.24  (-0.24) | | 0.28 (0.38) | -0.83  (-0.64) | 0.410 (0.52) |
| RALP/LARP* | -0.36  (-0.42) | 0.34 (0.43) | -1.07  (-0.98) | 0.289 (0.331) | 0.67 (0.44) | | 0.71 (0.51) | 0.95 (0.87) | 0.345 (0.386) |
| Roll tilt | 0.69 (0.78) | 0.67 (0.65) | 1.02 (1.20) | 0.310 (0.235) | 0.49 (0.48) | | 0.41 (0.65) | 1.19 (0.74) | 0.236 (0.460) |
| y-translation | **1.12 (1.07)** | **0.33 (0.34)** | **3.42 (3.18)** | **0.001 (0.002)** | 0.02 (0.28) | | 0.21 (0.34) | 0.12 (0.82) | 0.908 (0.413) |
| z-translation | -0.083 (0.24) | 0.210 (0.32) | -0.40 (0.74) | 0.692 (0.464) | 0.19  (-0.02) | | 0.42 (0.32) | 0.45  (-0.07) | 0.653 (0.946) |
| Intercept | 4.71 (4.68) | 0.53 (0.30) | 8.83 (15.72) | <0.001 (<0.001) | 4.91 (6.10) | | 0.55 (0.42) | 8.88 (14.19) | <0.001  (<0.001) |
| **Condition 3:** Eyes Open, Airex Foam Surface (n=88) | | | | | | | | | |
| Yaw | -0.72  (-0.99) | 0.46 (0.62) | -1.570  (-1.62) | 0.121 (0.110) | **-0.85**  (-1.0) | | **0.36** (0.56) | **-2.36**  (-1.79) | **0.021**  (0.078) |
| RALP/LARP* | 0.17  (-0.23) | 0.39 (.45) | 0.43  (-0.50) | 0.667 (0.617) | 0.87  (-0.48) | | 0.52 (0.54) | 1.66  (-0.09) | 0.100 (0.37) |
| Roll tilt | 0.68 (1.0) | 0.60 (0.70) | 1.13 (1.44) | 0.263 (0.152) | **1.54 (1.04)** | | **0.52 (0.72)** | **2.97 (2.65)** | **0.004**  **(0.010)** |
| y-translation | **1.53 (1.13)** | **0.63 (0.40)** | **2.41 (2.81)** | **0.018 (0.006)** | -0.20 (1.01) | | 0.27 (0.38) | -0.74 (1.69) | 0.464 (0.095) |
| z-translation | -0.27 **(0.80)** | 0.20 **(0.38)** | -1.39 **(2.14)** | 0.169  (**0.035)** | -0.13 (.039) | | 0.39 (0.38) | -0.34 (1.13) | 0.731 (0.261) |
| Intercept | 6.48 (6.90) | 0.49 (0.41) | 13.37 (16.81) | <0.001 (<0.001) | 7.24 (7.82) | | 0.64 (0.38) | 11.40 (20.52) | <0.001  (<0.001) |

**Supplemental Table 8**. Full regression analyses for Conditions 1, 2, & 3 assessing ML RMS and AP RMS to each age- adjusted threshold. Results from analyses using non-age adjusted thresholds are shown parenthetically. Values which are statistically significant (i.e. p ≤ 0.05) are bolded.

|  | **ML RMS** | | | | **AP RMS** | | | |
| --- | --- | --- | --- | --- | --- | --- | --- | --- |
|  | β | SE | t | p-value | β | SE | t | p-value |
| **Condition 1:** Eyes Open, Firm Surface (n=98) | | | | | | | | |
| Yaw | 0.05 (0.30) | 0.26 (0.33) | 0.18 (0.30) | >0.99  (>0.99) | 0.05 (0.26) | 0.24 (0.32) | 0.20 (0.83) | >0.99  (>0.99) |
| RALP/LARP* | -0.18 (0.18) | 0.23 (0.27) | -0.77 (0.68) | >0.99  (>0.99) | -0.28 (-0.23) | 0.37 (0.43) | -0.78 (-0.54) | >0.99  (>0.99) |
| Roll tilt | 0.52 (0.91) | 0.42 (0.37) | 1.23 (2.48) | >0.99  (0.240) | 0.55 (0.49) | 0.39 (0.40) | 1.41 (1.21) | >0.99  (>0.99) |
| y-translation | 0.71 (0.70) | 0.29 (0.24) | 2.43 (2.87) | 0.085  (0.075) | 0.21 (0.02) | 0.54 (0.46) | 0.38  (0.04) | >0.99  (>0.99) |
| z-translation | 0.23 **(0.65)** | 0.21 **(0.21)** | 1.11 **(3.11)** | >0.99  (**0.030)** | -0.15 (-0.13) | 0.25 (0.35) | -0.61 (-0.38) | >0.99  (>0.99) |
| **Condition 2:** Eyes Closed, Firm Surface (n=98) | | | | | | | | |
| Yaw | 0.39 (0.73) | 0.34 (0.43) | 1.13 (0.69) | >0.99  (>0.99) | 0.44 (0.97) | 0.34 (0.41) | 1.29 (2.38) | >0.99  (0.30) |
| RALP/LARP* | 0.02 (0.50) | 0.26 (0.32) | 0.07  (1.53) | >0.99  (>0.99) | 0.36 (0.70) | 0.30 (0.44) | 1.19 (2.25) | >0.99  (0.41) |
| Roll tilt | 1.03 (1.57) | 0.55 (0.53) | 1.89 (2.91) | 0.310  (0.062) | 1.13 (1.48) | 0.51 (0.41) | 2.22 (3.10) | 0.144  (0.054) |
| y-translation | **1.19**  **(1.25)** | **0.37 (0.31)** | **3.24**  **(4.04)** | **0.010**  **(0.001)** | 0.71 (0.95) | 0.44 (0.47) | 1.62 (2.79) | 0.541 (0.09) |
| z-translation | -0.01 **(1.10)** | 0.205 **(0.33)** | -0.08 **(3.19)** | >0.99  (**0.031**) | 0.01 **(0.87)** | 0.21 **(0.29)** | 0.06 **(3.00)** | >0.99  (**0.045**) |
| **Condition 3:** Eyes Open, Airex Foam Surface (n=95) | | | | | | | | |
| Yaw | 0.10 (0.56) | 0.43 (0.44) | 0.22 (1.26) | >0.99  (>0.99) | -0.13 (0.12) | 0.34 (0.39) | -0.38 (0.10) | >0.99  (>0.99) |
| RALP/LARP* | 0.25  (1.0) | 0.43 (0.40) | 0.57 (2.62) | >0.99  (0.150) | -0.07 (0.40) | 0.44 (0.41) | -0.16 (0.97) | >0.99  (>0.99) |
| Roll tilt | 1.00 (1.82) | 0.45 (0.45) | 2.23 (4.04) | 0.14  **(0.001)** | 0.84 (1.15) | 0.41 (0.43) | 2.07 (2.68) | 0.205  (0.135) |
| y-translation | **1.46 (1.51)** | **0.56 (0.28)** | **2.64**  **(5.51)** | **0.049**  **(<0.001)** | **1.25 (0.91)** | **0.43 (0.28)** | **2.88 (3.20)** | **0.025**  **(0.030)** |
| z-translation | -0.17  **(1.41)** | 0.18 **(0.30)** | -0.94  **(4.68)** | >0.99  **(<0.001**) | -0.05 (0.72) | 0.24 (0.29) | -0.18 (2.50) | >0.99  (0.21) |

**Supplemental Table 9.** Univariable regression analyses assessing the relationship between ML RMS and each age-adjusted threshold during Condition 1, 2, and 3. Results from logarithmic thresholds are presented in parentheticals. P-values represent corrected p-values using a Bonferroni adjustment applied across each plane for all three conditions (i..e., corrected p = p*15). *N=89; **N=88. Values which are statistically significant (i.e. p ≤ 0.05) are bolded.

|  | **ML Mean Velocity** | | | | **AP Mean Velocity** | | | |
| --- | --- | --- | --- | --- | --- | --- | --- | --- |
|  | **β** | **SE** | **t** | **p-value** | **β** | **SE** | **t** | **p-value** |
| **Condition 1:** Eyes Open, Firm Surface (n=89) | | | | | | | | |
| Yaw | 2.50  (-0.57) | 3.34  (0.97) | 0.75  (-0.59) | 0.457  (0.558) | 0.43  (-0.27) | 1.43  (1.11) | 0.20  (-0.25) | 0.766  (0.806) |
| RALP/LARP | -1.64  (-0.98) | 1.07  (0.92) | -1.53  (-1.06) | 0.130  (0.292) | -0.38  (-0.61) | 1.16  (1.00) | -0.33  (-0.61) | 0.746  (0.543) |
| Roll tilt | 0.40  (1.19) | 1.70  (1.46) | 0.24  (0.82) | 0.814  (0.417) | 2.38  (2.10) | 2.29  (2.07) | 1.04  (1.01) | 0.301  (0.316) |
| y-translation | **2.39**  (2.02) | **1.07**  (0.99) | **2.23**  (2.04) | **0.029**  (0.44) | 2.10  **(2.22)** | 1.13  **(1.10)** | 1.85  **(2.05)** | 0.068  **(0.043)** |
| z-translation | **-1.67**  **(-1.08)** | **0.73**  **(.45)** | **-2.29**  **(-2.38)** | **0.025**  **(0.020)** | **-1.35**  (0.62) | **0.63**  (1.01) | **-2.13**  (0.61) | **0.037**  (0.543) |
| Intercept | 10.18  (10.33) | 1.40 (1.38) | 7.30  (7.46) | 0.001 (<0.001) | 7.49  (9.68) | 1.94  (1.43) | 3.86  (6.76) | 0.001 (<0.001) |
| **Condition 2:** Eyes Closed, Firm Surface (n=89) | | | | | | | | |
| Yaw | 3.63  (1.43) | 3.31  (2.80) | 1.10  (0.51) | 0.275  (0.609) | 2.04  (-0.24) | 2.63  (2.20) | 0.78  (-0.11) | 0.440  (0.912) |
| RALP/LARP | **-3.03**  (-2.57) | **1.46**  (2.11) | **-2.07**  (-1.22) | **0.041**  (0.227) | 0.10  (-1.25) | 2.28  (1.87) | 0.05  (-0.66) | 0.964  (0.508) |
| Roll tilt | 1.56  (1.95) | 3.11  (3.20) | 0.50  (0.61) | 0.616  (0.543) | -0.16  (0.21) | 2.55  (2.57) | -0.06  (0.09) | 0.949  (0.932) |
| y-translation | 3.85  **(3.98)** | 2.07  **(1.97)** | 1.86  **(2.01)** | 0.066  (**0.047)** | 2.74  (2.14) | 2.07  (1.88) | 1.32  (1.14) | 0.189  (0.257) |
| z-translation | **-2.30**  (2.18) | **0.96**  (1.83) | **-2.39**  (1.20) | **0.019**  (0.235) | -1.40  **(4.51)** | 0.84  **(1.69)** | -1.67  **(2.66)** | 0.098  (**0.009**) |
| Intercept | 13.69  (15.03) | 2.51  (2.21) | 5.45  (6.80) | 0.001 (<0.001) | 12.60  (11.04) | 2.45  (1.68) | 5.07  (6.58) | 0.001 (<0.001) |
| **Condition 3:** Eyes Open, Airex Foam Surface (n=88) | | | | | | | | |
| Yaw | -0.68  (-2.98) | 2.53  (2.53) | -0.27  (-1.16) | 0.788  (0.251) | -2.65  (-2.83) | 2.07  (2.20) | -0.32  (-1.37) | 0.753  (0.176) |
| RALP/LARP | 1.71  (0.07) | 2.14  (2.28) | 0.80  (0.03) | 0.427  (0.973) | 1.14  (0.35) | 2.12  (2.39) | 0.54  (0.15) | 0.594  (0.883) |
| Roll tilt | 0.94  (2.98) | 2.71  (3.23) | 0.35  (0.92) | 0.728  (0.359) | 3.38  (4.32) | 3.22  (3.42) | 1.05  (1.27) | 0.296  (0.209) |
| y-translation | 3.21  (4.10) | 1.99  (2.40) | 1.61  (1.71) | 0.111  (0.091) | **5.87**  **(4.37)** | **2.62**  **(2.08)** | **2.24**  **(2.11)** | **0.028**  (**0.038**) |
| z-translation | **-2.62**  (2.59) | **0.86**  (1.89) | **-3.05**  (1.37) | **0.003**  (0.174) | **-3.54**  (2.46) | **1.01**  (1.77) | **-3.52**  (1.39) | **0.001**  (0.167) |
| Intercept | 19.09  18.50 | 2.23  (2.34 | 8.56  (7.89) | 0.001 (<0.001) | 15.38  (17.43) | 2.75  (2.31) | 5.57  (7.52) | 0.001 (<0.001) |

**Supplemental Table 10**. Full regression analyses for Conditions 1-3 of assess ML Mean Velocity (MV) and AP MV to each age- adjusted threshold. Results from logarithmic thresholds are presented in parentheticals. Values which are statistically significant (i.e. p ≤ 0.05) are bolded.

|  | **ML Mean Velocity** | | | | | **AP Mean Velocity** | | | |
| --- | --- | --- | --- | --- | --- | --- | --- | --- | --- |
|  | **β** | **SE** | **t** | **p-value** | **β** | | **SE** | **t** | **p-value** |
| **Condition 1:** Eyes Open, Firm Surface (n=98) | | | | | | | | | |
| Yaw | 1.19  (1.92) | 0.92  (0.92) | 1.30  (2.10) | 0.976  (0.580) | 2.25  (2.38) | | 1.50  (1.12) | 1.51  (2.11) | >0.99  (0.557) |
| RALP/LARP* | -0.39  (0.29) | 0.63  (0.90) | -0.30  (1.58) | >0.99  (>0.99) | 0.34  (2.11) | | 0.85  (1.02) | 0.40  (2.07) | >0.99  (0.626) |
| Roll tilt | 0.25  (2.45) | 1.32  (0.79) | 0.19  (2.81) | >0.99  (0.090) | 2.31  (3.72) | | 2.07  (1.32) | 1.12  (2.82) | >0.99  (0.088) |
| y-translation | 1.48  (2.69) | 0.92  (0.79) | 1.60  (3.14) | 0.561  (0.143) | 2.44  **(2.99)** | | 1.41  **(0.87)** | 1.73  **(3.45)** | 0.861  (**0.013)** |
| z-translation | **-1.40**  **(2.26)** | **0.47**  **(0.59)** | **-2.96**  **(3.81)** | **0.020**  **(0.003)** | -1.16  **(2.28)** | | 0.55  **(0.49)** | -2.08  **(4.61)** | 0.406  (<**0.001**) |
| **Condition 2:** Eyes Closed, Firm Surface (n=98) | | | | | | | | | |
| Yaw | 5.79  (5.58) | 2.59  (1.94) | 2.23  (2.87) | 0.279  (0.080) | 4.86  (5.18) | | 2.29  (2.02) | 2.12  (2.56) | 0.365  (0.358) |
| RALP/LARP* | -0.76  (3.25) | 1.25  (1.60) | -0.09  (2.03) | >0.99  (0.675) | 0.62  (3.45) | | 1.39  (1.40) | 0.45  (2.46) | >0.99  (0.474) |
| Roll tilt | 2.19  **(6.13)** | 2.69  **(1.84)** | 0.82  **(3.34)** | >0.99  (**0.018**) | 0.94  (5.71) | | 2.59  (1.89) | 0.36  (3.03) | >0.99  (0.096) |
| y-translation | 4.62  **(4.73)** | 2.46  **(1.48)** | 1.87  **(3.86)** | 0.644  (**0.030)** | 3.09  **(5.34)** | | 2.13  **(1.47)** | 1.45  **(3.63)** | >0.99  **(0.014)** |
| z-translation | **-2.64**  **(4.74)** | **0.90**  **(1.22)** | **-2.93**  **(3.89)** | **0.043**  **(0.003)** | -2.01  **(6.09)** | | 0.96  **(1.29)** | -2.10  **(4.71)** | 0.383  **(<0.001)** |
| **Condition 3:** Eyes Open, Airex Foam Surface (n=95) | | | | | | | | | |
| Yaw | 2.26  (2.93) | 2.11  (1.74) | 1.08  (1.69) | >0.99  (>0.99) | 4.39  (4.43) | | 2.18  (1.82) | 2.01  (2.43) | 0.473  (0.503) |
| RALP/LARP* | 0.83  (4.24) | 1.31  (1.55) | 0.64  (2.73) | >0.99  (0.225) | 1.83  (5.44) | | 1.62  (1.86) | 1.13  (2.92) | >0.99  (0.135) |
| Roll tilt | 0.89  **(6.15)** | 2.12  **(1.80)** | 0.42  **(3.42)** | >0.99  (**0.013**) | 3.34  (4.45) | | 3.11  (2.24) | 1.07  (2.77) | >0.99  (0.080) |
| y-translation | 3.37  **(5.68)** | 2.03  **(1.43)** | 1.66  **(3.98)** | 0.998  (**0.002**) | 6.25  **(6.79)** | | 2.69  **(1.48)** | 2.32  **(4.59)** | 0.227  **(<0.001**) |
| z-translation | **-2.87**  **(5.05)** | **0.80**  **(1.27)** | **-3.60**  **(3.97)** | **0.005**  **(0.002)** | **-3.34**  **(5.78)** | | **0.93**  **(1.14)** | **-3.59**  **(5.07)** | **0.005**  **(<0.001)** |

**Supplemental Table 11.** Univariable regression analyses assessing the relationship between ML MV and AP MV and each age-adjusted threshold during Condition 1, 2, and 3. Results from logarithmic thresholds are presented in parentheticals. P-values represent corrected p-values using a Bonferroni adjustment applied across each plane for all three conditions (i.e., corrected p = p*15). *N=89; **N=88. Values which are statistically significant (i.e. corrected p ≤ 0.05) are bolded.

|  | **ML Mean Frequency** | | | | **AP Mean Frequency** | | | |
| --- | --- | --- | --- | --- | --- | --- | --- | --- |
|  | **β** | **SE** | **t** | **p-value** | **β** | **SE** | **t** | **p-value** |
| **Condition 1:** Eyes Open, Firm Surface (n=89) | | | | | | | | |
| Yaw | 0.110  (0.051) | 0.080  (0.066) | 1.37  (0.78) | 0.175  (0.437) | 0.034  (0.015) | 0.029  (0.033) | 1.18  (0.46) | 0.243  (0.646) |
| RALP/LARP | -0.03  (-0.062) | 0.041  (0.054) | -0.74  (-1.15) | 0.459  (0.254) | 0.031  (0.033) | 0.036  (0.039) | 0.86  (0.85) | 0.393  (0.399) |
| Roll tilt | -0.060  (-0.512) | 0.048  (0.553) | -1.24  (-0.93) | 0.219  (0.357) | 0.033  (0.013) | 0.056  (0.062) | 0.58  (0.21) | 0.563  (0.832) |
| y-translation | 0.025  (0.055) | 0.046  (0.050) | 0.54  (1.11) | 0.592  (0.270) | -0.005  (0.008) | 0.031  (0.035) | -0.16  (0.23) | 0.876  (0.818) |
| z-translation | -0.037  **(0.106)** | 0.028  **(0.032)** | -1.35  **(3.24)** | 0.181  (**0.002**) | -0.062  (0.001) | 0.018  (0.033) | -1.39  (0.05) | 0.100  (0.962) |
| Intercept | 0.453  (0.360) | 0.062  (0.044) | 7.33  (8.16) | 0.001 (<0.001) | 0.39  (0.398) | 0.056  (0.042) | 6.93  (9.33) | 0.001 (<0.001) |
| **Condition 2:** Eyes Closed, Firm Surface (n=89) | | | | | | | | |
| Yaw | 0.132  (0.075) | 0.079  (0.077) | 1.67  (0.97) | 0.100  (0.333) | 0.081  (0.031) | 0.064  (0.051) | 1.27  (0.62) | 0.208  (0.540) |
| RALP/LARP | -0.113  (-0.116) | 0.039  (0.063) | -1.90  (-1.85) | 0.053  (0.069) | 0.068  (0.023) | 0.088  (0.065) | 0.77  (0.36) | 0.441  (0.719) |
| Roll tilt | -0.041  (-0.006) | 0.06  (0.071) | -0.69  (-0.09) | 0.492  (0.930) | 0.029  (0.038) | 0.106  (0.102) | 0.27  (0.38) | 0.786  (0.707) |
| y-translation | 0.075  (0.095) | 0.058  (0.052) | 1.29  (1.82) | 0.201  (0.073) | 0.025  (-0.006) | 0.064  (0.054) | 0.40  (-0.11) | 0.692  (0.911) |
| z-translation | **-0.068**  (0.067) | **0.026**  (0.046) | **-2.66**  (1.45) | **0.009**  (0.152) | -0.074  (0.078) | 0.032  (0.059) | -2.34  (1.45) | 0.022  (0.150) |
| Intercept | 0.593  (0.505) | 0.065  (0.054) | 9.15  (9.22) | 0.001 (<0.001) | 0.453  (0.461) | 0.102  (0.070) | 4.43  (6.56) | 0.001 (<0.001) |
| **Condition 3:** Eyes Open, Airex Foam Surface (n=88) | | | | | | | | |
| Yaw | 0.021  (0.005) | 0.057  (0.052) | 0.37  (0.10) | 0.710  (0.923) | 0.024  (0.003) | 0.030  (0.033) | 0.79  (0.11) | 0.433  (0.911) |
| RALP/LARP | 0.024  (0.026) | 0.046  (0.051) | 0.52  (0.52) | 0.601  (0.606) | 0.010  (0.023) | 0.043  (0.049) | 0.24  (0.49) | 0.812  (0.626) |
| Roll tilt | -0.022  (-0.002) | 0.048  (0.054) | -0.47  (-0.05) | 0.638  (0.964) | 0.025  (0.032) | 0.056  (0.058) | 0.45  (0.56) | 0.653  (0.578) |
| y-translation | 0.002  (0.025) | 0.051  (0.056) | 0.04  (0.45) | 0.966  (0.656) | 0.047  (0.020) | 0.038  (0.034) | 1.23  (0.59) | 0.222  (0.555) |
| z-translation | **-0.05**  (0.048) | **0.024**  (0.041) | **-2.05**  (1.20) | **0.044**  (0.235) | **-0.072**  (0.020) | **0.019**  (0.034) | **-3.87**  (0.59) | **0.001**  (0.556) |
| Intercept | 0.553  (0.480) | 0.056  (0.052) | 9.96  (9.50) | 0.001 (<0.001) | 0.504  (0.490) | 0.054  (0.043) | 9.31  (11.25) | 0.001 (<0.001) |

**Supplemental Table 12**. Full regression analyses for Condition 1-3 assessing ML mean frequency (MF) and AP MF to each age-adjusted threshold. Results from logarithmic thresholds are presented in parentheticals. Values which are statistically significant (i.e. p ≤ 0.05) are bolded.

|  | **ML Mean Frequency** | | | | | **AP Mean Frequency** | | | | |
| --- | --- | --- | --- | --- | --- | --- | --- | --- | --- | --- |
|  | β | SE | t | p-value | β | | SE | t | p-value |  |
| **Condition 1:** Eyes Open, Firm Surface (n=98) | | | | | | | | | | |
| Yaw | 0.102  (0.086) | 0.061  (0.049) | 1.67  (1.75) | 0.986  (>0.99) | 0.062  (0.081) | | 0.037  (0.039) | 1.70  (2.07) | 0.918  (0.620) |  |
| RALP/LARP* | -0.015  (0.054) | 0.027  (0.036) | -0.58  (1.50) | >0.99  (>0.99) | 0.023  (0.054) | | 0.027  (0.029) | 0.85  (1.86) | >0.99  (>0.99) |  |
| Roll tilt | -0.049  (0.048) | 0.040  (0.036) | -1.22  (1.32) | >0.99  (>0.99) | 0.002  (0.076) | | 0.057  (0.038) | 0.04  (2.00) | >0.99  (0.722) |  |
| y-translation | 0.044  (0.086) | 0.053  (0.038) | 0.84  (1.23) | >0.99  (>0.99) | 0.012  (0.067) | | 0.038  (0.031) | 0.31  (2.16) | >0.99  (0.500) |  |
| z-translation | -0.051  (0.091) | 0.025  (0.091) | -2.00  (2.28) | 0.488  (0.741) | **-0.075**  (0.058) | | **0.021**  (0.025) | **-3.61**  (2.38) | **0.005**  **(**0.287) |  |
| **Condition 2:** Eyes Closed, Firm Surface (n=98) | | | | | | | | | | |
| Yaw | 0.118  (0.089) | 0.065  (0.053) | 1.81  (1.67) | 0.740  (>0.99) | 0.128  (0.131) | | 0.061  (0.056) | 2.08  (2.35) | 0.400  (0.316) |  |
| RALP/LARP* | -0.041  (0.028) | 0.037  (0.040) | -1.11  (0.07) | >0.99  (>0.99) | 0.062  (0.116) | | 0.061  (0.055) | 1.02  (2.09) | >0.99  (0.588) |  |
| Roll tilt | -0.051  (0.042) | 0.049  (0.041) | -1.04  (1.02) | >0.99  (>0.99) | 0.021  (0.106) | | 0.096  (0.065) | 0.22  (1.64) | >0.99  (>0.99) |  |
| y-translation | 0.076  (0.089) | 0.062  (0.038) | 1.21  (2.31) | >0.99  (0.342) | 0.051  (0.092) | | 0.070  (0.045) | 0.72  (2.02) | >0.99  (0.699) |  |
| z-translation | -0.081  (0.064) | 0.025  (0.064) | -3.28  (2.09) | 0.014  (0.593) | -0.093  (0.115) | | 0.033  (0.036) | -2.86  (3.17) | 0.053  (0.060) |  |
| **Condition 3:** Eyes Open, Airex Foam Surface (n=95) | | | | | | | | | | |
| Yaw | 0.031  (0.067_ | 0.041  (0.034) | 0.78  (2.00) | >0.99  (>0.99) | 0.071  (0.073) | | 0.034  (0.028) | 2.10  (2.63) | 0.387  (0.148) |  |
| RALP/LARP** | 0.036  (0.079) | 0.031  (0.031) | 1.16  (2.56) | >0.99  (0.363) | 0.035  (0.069) | | 0.029  (0.031) | 1.22  (2.20) | >0.99  (0.460) |  |
| Roll tilt | -0.026  (0.069) | 0.043  (0.033) | -0.60  (2.11) | >0.99  (>0.99) | 0.030  (0.082) | | 0.055  (0.036) | 0.54  (2.20) | >0.99  (0.466) |  |
| y-translation | 0.009  (0.076) | 0.043  (0.034) | 0.22  (2.24) | >0.99  (0.821) | 0.055  (0.063) | | 0.042  (0.025) | 1.30  (2.52) | >0.99  (0.199) |  |
| z-translation | -0.063  (0.078) | 0.023  (0.034) | -2.76  (3.30) | 0.071  (0.051) | **-0.076**  (0.054) | | **0.017**  (0.018) | **-4.38**  (2.95) | **<0.001**  (0.061) |  |

**Supplemental Table 13**. Univariable regression analyses assessing the relationship between ML and AP MV and each age-adjusted threshold during Condition 1, 2, and 3. Results from logarithmic thresholds are presented in parentheticals-values represent corrected p-values using a Bonferroni adjustment applied across each plane for all three conditions (i.e., corrected p = p*15). *N=89; **N=88. Values which are statistically significant (i.e. corrected p ≤ 0.05) are bolded.
